# Lifestyle factors and hand eczema: A systematic review and meta‐analysis of observational studies

**DOI:** 10.1111/cod.14102

**Published:** 2022-04-02

**Authors:** Laura Loman, Marjolein J. Brands, Anna A. L. Massella Patsea, Klaziena Politiek, Bernd W. M. Arents, Marie L. A. Schuttelaar

**Affiliations:** ^1^ Department of Dermatology University of Groningen, University Medical Center Groningen Groningen The Netherlands; ^2^ Department of Dermatology Medical Center Leeuwarden Leeuwarden The Netherlands; ^3^ Dutch Association for People with Atopic Dermatitis Nijkerk The Netherlands

**Keywords:** alcohol consumption, BMI, hand dermatitis, hand eczema, lifestyle, physical activity, smoking, stress

## Abstract

Evidence regarding the association between lifestyle factors and hand eczema is limited.To extensively investigate the association between lifestyle factors (smoking, alcohol consumption, stress, physical activity, body mass index, diet, and sleep) and the prevalence, incidence, subtype, severity, and prognosis of hand eczema, a systematic review and meta‐analysis were conducted in accordance with the Meta‐analysis Of Observational Studies in Epidemiology consensus statement. MEDLINE, Embase, and Web of Science were searched up to October 2021. The (modified) Newcastle‐Ottawa Scale was used to judge risk of bias. Quality of the evidence was rated using the Grades of Recommendation, Assessment, Development and Evaluation approach. Eligibility and quality were blindly assessed by two independent investigators; disagreements were resolved by a third investigator. Data were pooled using a random‐effects model, and when insufficient for a meta‐analysis, evidence was narratively summarized. Fifty‐five studies were included. The meta‐analysis (17 studies) found very low quality evidence that smoking is associated with a higher prevalence of hand eczema (odds ratio 1.18, 95% confidence interval 1.09‐1.26). No convincing evidence of associations for the other lifestyle factors with hand eczema were found, mostly due to heterogeneity, conflicting results, and/or the limited number of studies per outcome.

## Graphical abstract

- This extensive systematic review and meta‐analysis found very low quality evidence that smoking is associated with the prevalence of hand eczema.
- Due to the limited number of studies per outcome, heterogeneity, and/or conflicting results, no firm conclusions can be drawn regarding other lifestyle factors.
- However, the limited available evidence may suggest a positive association between stress, BMI, and prevalence of hand eczema, and a possible role for stress as aggravating factor of hand eczema.

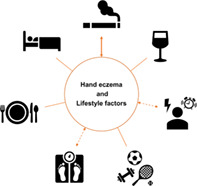

## 1 INTRODUCTION

Hand eczema (HE) is a common skin disease with a considerable patient burden regarding psychosocial and economic consequences.^1^, ^2^ Current risk factors for HE are classified as exogenous and endogenous, with atopic dermatitis (AD)^3^ as the most well‐known endogenous risk factor, and exposure to irritants (wet work, friction)^4^, ^5^ and contact allergens^6^ as exogenous factors. Its etiology is complex and multifactorial, including genetic predisposition, immune dysfunction, and environmental factors.

Lifestyle has been the subject of increased research for preventing disease and improving health and well‐being. For skin diseases like AD^7^, ^8^, ^9^ and psoriasis,^10^ there is already increased awareness of lifestyle factors and modifiable behavior. However, little is known about the association between lifestyle factors and HE. It could be hypothesized that lifestyle factors such as smoking^11^ and stress^12^ may skew the immune system toward T helper (Th) 2 immunity, which could, at least in theory, increase the occurrence, severity, and/or worsen the prognosis of HE. Likewise, obesity is associated with a chronic low‐grade inflammatory state, which might also influence HE.^13^ In addition, for smoking, also non‐immunologic effects such as cutaneous vasoconstriction with delayed wound healing might play a role in the severity and/or prognosis of HE.^14^ Following this hypothesis, it is possible that more inflammatory subtypes such as vesicular HE, or HE accompanied by AD, are more influenced by lifestyle factors compared to other subtypes. The aim of this systematic review and meta‐analysis was to assess the association between lifestyle factors (including smoking, alcohol consumption, stress, physical activity, body mass index [BMI], diet, and sleep) and prevalence, incidence, subtype, severity, and prognosis of HE. Because lifestyle factors relate to human behavior and exposing subjects to unfavorable conditions might be harmful and unethical, this study focused on observational studies including subjects with all subtypes of HE.

## 2 METHODS

This study was conducted in accordance with the Meta‐analysis Of Observational Studies in Epidemiology (MOOSE) consensus statement.^15^, ^16^ A protocol was registered prospectively in PROSPERO (International Prospective Register of Systematic Reviews) (CRD42020207731).

### 2.1 Data sources

A literature search was conducted in MEDLINE, Embase, and Web of Science from inception to October 14, 2021, supervised by an experienced research librarian. Search terms included all terms regarding HE and synonyms, combined with synonymous and related terms for lifestyle factors (see Appendix S1 in the supplement for the full search strategy).

### 2.2 Study selection

After de‐duplication,^17^ all studies were uploaded in RAYYAN (http://rayyan.qcri.or/)^18^ for blinded and independent screening for eligibility based on title, abstract, and keywords by two investigators (LL, MJB). Disagreements were resolved by reaching consensus or otherwise treated as a provisional inclusion awaiting full text. References of included studies and possibly relevant reviews were searched manually for additional studies. Broad inclusion criteria were applied, and all human studies that assessed the association between lifestyle factors and HE, regardless of the underlying etiology, were included. Excluded were studies without primary data, case reports, case series (n < 10), reviews, studies that only assessed the association between HE and second‐hand smoking, tobacco allergy, skin exposure and food (substances), or the use of topical alcohol as disinfectant. We applied no language restrictions.

Subsequently, full texts were retrieved of all the (provisionally) included studies. Studies published in languages other than English, Dutch, or German, were translated by colleagues with sufficient knowledge of the particular language. Abstracts from unpublished studies were also included if sufficient data were provided. Multiple papers from a single study were included if each presented unique data. Final inclusion was assessed independently by two authors (LL, MJB), and any disagreements were resolved by consulting a third author (BWMA).

### 2.3 Data extraction

From each included study, first author name, publication year, country, study setting and design, number of total subjects, and number of subjects with HE were extracted. For study outcomes, assessment of HE, outcome ascertainment, instruments used, and primary study outcomes were recorded. A list of excluded studies based on full text, including justifications, was maintained. Authors were contacted in case of insufficient information or in case full text was not available.

### 2.4 Assessment of risk of bias and overall quality of the evidence

We used the Newcastle‐Ottawa Scale (NOS) for cohort and case–control studies for quality assessment.^19^ An adapted version of the NOS was used for cross‐sectional studies, as per Quaade et al.^2^ Each study was assessed independently in pairs of two among three authors (LL, MJB, AALMP); a fourth author (BWMA) was asked to resolve differences. If studies reported results on multiple lifestyle factors and/or outcome measures, quality assessment was conducted per reported outcome measure. Cohort and case–control study outcomes with ≥6 points on the NOS, and cross‐sectional study outcomes with ≥7 points, were considered as low risk of bias. To easily identify the NOS items that were low (one or two stars) or high risk of bias (zero stars), we used Cochrane's RevMan version 5.4.1.^20^ for a visual presentation, as was done previously by Papola et al.^21^ Of the three NOS scales used (case–control, cohort, and cross‐sectional) we divided the item comparability in two (sex and age) so that these are also easily identifiable. For the meta‐analyses the Grades of Recommendation, Assessment, Development and Evaluation (GRADE)^22^ methodology was used to assess the overall quality of the evidence (high, moderate, low, or very low).

### 2.5 Data synthesis

In case of missing summary statistics, odds ratios (ORs) were calculated from raw data, if possible. From the studies included in the meta‐analyses, forest plots with estimated pooled ORs with 95% confidence intervals (CIs) were generated using Cochrane's RevMan version 5.4.1.^20^ We chose the random‐effects model for meta‐analyses as the variability between studies was assumed to be high. Overlap of CIs or point estimates were used to judge inconsistency. To explore the possible causes of heterogeneity among study results, a subgroup analysis based on setting (occupational vs non‐occupational) was performed. To assess the robustness of the synthesized results, a sub‐analysis was performed including only studies with an overall low risk of bias. If the number of studies allowed it, funnel plot visualization was used to inspect potential publication bias. When data were insufficient to conduct a meta‐analysis, the evidence was narratively summarized.

## 3 RESULTS

### 3.1 Literature search

**FIGURE 1:**
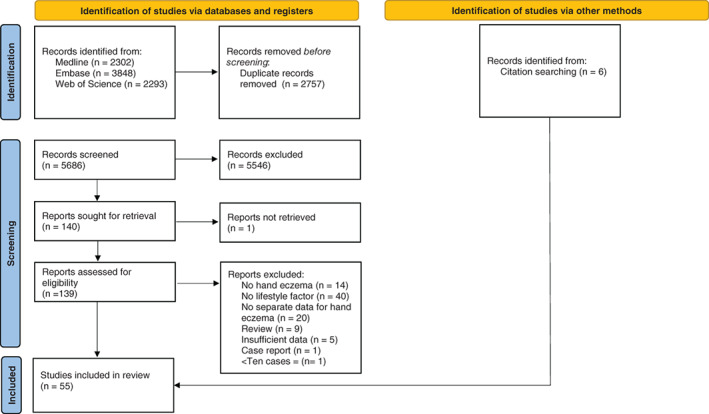
Preferred Reporting Items for Systematic Reviews and Meta‐Analyses (PRISMA) 2020 flow diagram for new systematic reviews, which included searches of databases, registers, and other sources

The search identified 5686 records for screening after de‐duplication,^17^ of which 140 met the criteria for full‐text extraction. Of these, 91 were excluded, based mainly on not reporting (separate) data for HE or lifestyle factors (see Table S2 in the Supplement for a list of excluded studies with justification). Six studies were included from references. See Figure 1 for the Preferred Reporting Items for Systematic Reviews and Meta‐Analyses (PRISMA) 2020 flowchart.^15^

### 3.2 Study characteristics

A total of 55 studies (n = 40‐65 261),^1^, ^5^, ^23^, ^24^, ^25^, ^26^, ^27^, ^28^, ^29^, ^30^, ^31^, ^32^, ^33^, ^34^, ^35^, ^36^, ^37^, ^38^, ^39^, ^40^, ^41^, ^42^, ^43^, ^44^, ^45^, ^46^, ^47^, ^48^, ^49^, ^50^, ^51^, ^52^, ^53^, ^54^, ^55^, ^56^, ^57^, ^58^, ^59^, ^60^, ^61^, ^62^, ^63^, ^64^, ^65^, ^66^, ^67^, ^68^, ^69^, ^70^, ^71^, ^72^, ^73^, ^74^, ^75^ comprising more than 42 500 individuals with HE on 98 different lifestyle factors and/or outcome measures, were included (Table 1). Study design included cohort (prospective (n = 13)^23^, ^24^, ^29^, ^36^, ^37^, ^38^, ^51^, ^52^, ^56^, ^58^, ^69^, ^74^, ^75^ and retrospective (n = 6)^27^, ^44^, ^55^, ^57^, ^61^, ^62^), case–control (n = 7),^1^, ^25^, ^39^, ^46^, ^53^, ^64^, ^71^ and cross‐sectional studies (n = 29).^5^, ^26^, ^28^, ^30^, ^31^, ^32^, ^33^, ^34^, ^35^, ^40^, ^41^, ^42^, ^43^, ^45^, ^47^, ^48^, ^49^, ^50^, ^54^, ^59^, ^60^, ^63^, ^65^, ^66^, ^67^, ^68^, ^70^, ^72^, ^73^ HE was assessed in different study settings, including clinical (n = 15),^1^, ^23^, ^29^, ^44^, ^53^, ^54^, ^61^, ^64^, ^66^, ^67^, ^68^, ^69^, ^71^, ^74^, ^75^ occupational (n = 22),^5^, ^24^, ^25^, ^27^, ^30^, ^36^, ^40^, ^45^, ^47^, ^49^, ^50^, ^52^, ^55^, ^56^, ^57^, ^58^, ^59^, ^60^, ^62^, ^63^, ^65^, ^73^ and the general population (n = 18).^26^, ^28^, ^31^, ^32^, ^33^, ^34^, ^35^, ^37^, ^38^, ^39^, ^41^, ^42^, ^43^, ^46^, ^48^, ^51^, ^70^, ^72^ The diagnoses of HE was either retrieved from medical records (n = 5),^23^, ^44^, ^55^, ^61^, ^62^ self‐reported (n = 24),^5^, ^26^, ^27^, ^28^, ^30^, ^31^, ^32^, ^33^, ^34^, ^35^, ^37^, ^39^, ^40^, ^41^, ^43^, ^45^, ^46^, ^49^, ^50^, ^51^, ^53^, ^59^, ^70^, ^73^ or physician diagnosed (n = 23),^1^, ^24^, ^25^, ^29^, ^42^, ^47^, ^48^, ^54^, ^56^, ^57^, ^58^, ^60^, ^63^, ^64^, ^65^, ^66^, ^67^, ^68^, ^69^, ^71^, ^72^, ^74^, ^75^ or a combinations of self‐reported and physician diagnoses (n = 3).^36^, ^38^, ^52^ For the association between smoking and prevalence of HE a meta‐analysis could be performed. No meta‐analysis was possible for all other lifestyle factors and/or outcome measures due to heterogeneous methods and/or outcomes, and/or insufficient reporting of data. See Figure S3 for the risk of bias assessment of all outcomes.

**TABLE 1:** Details of included studies on lifestyle factors and hand eczema

Author	Year	Country	Study design	Setting	N (HE)	N (total)	Assessment of HE	Included subtype(s) of hand eczema as reported in the study	Assessment of lifestyle factor	Conclusion reported in article	NOS score
**Smoking**											
**Prevalence**											
Edman^23^	1988	Sweden	Prospective cohort	Clinical	153	425	Medical records	Vesicular palmar eczema	Yes/no	Smoking was positively associated with HE in males (*P* = 0.029, OR 2.4).	5
Uter^24^	1995	Germany	Prospective cohort	Occupational (hairdressing apprentices)	126	859	Physician diagnosed	HE (pompholyx or eczema)	>5 cigarettes/≤5 cigarettes daily	Smoking was positively associated with HE (OR 1.92, 95% CI: 1.27‐2.91).	5
Berndt^25^	2000	Switzerland	Prospective cohort with a nested case‐control	Occupational (metalworker trainees)	47	201	Physician diagnosed	HE not specified	Number of cigarettes	No association between smoking amount and HE.	4
Montnémery^26^	2005	Sweden	Cross‐sectional	General population	593^†^	9316	Self‐reported	HE not specified	>5 cigarettes/≤5 cigarettes daily	Smoking was positively associated with HE in the last year (OR 1.35, 95% CI: 1.04‐1.75).	8
Lind^27^	2006	Sweden	Retrospective cohort	Occupational/general population (hairdressers, controls from general population)	1068	8699	Self‐reported	HE not specified	Never/former/current	Smoking was positively associated with HE in hairdressers but not in controls (hairdressers: Current/former smokers: 31% and never‐smokers: 27% HE (*P* = 0.017); controls: Current/former smokers: 20% and never‐smokers: 18% HE (*P* = 0.054)).	5
Bø^28^	2008	Norway	Cross‐sectional	General population	1096	18 747	Self‐reported	HE not specified	Never/former/current + amount	No association between HE and smoking (current (m): OR 0.97, 95% CI 0.68‐1.38 previous (m): OR 0.99, 95% CI 0.73‐1.36 compared to never smoking. ≥20 cigarettes daily (m): OR 1.07, 95% CI: 0.73‐1.56. Current (f): OR 0.92, 95% CI 0.73‐1.16 Previous (f): OR 1.03, 95% CI 0.83‐1.28, ≥20 cigarettes daily (f): OR 1.20, 95% CI 0.87‐1.65).	6
Veien^29^	2008	Denmark	Prospective cohort	Private dermatological practice	522	522	Physician diagnosed	HE not specified	Yes/no	No association between smoking and HE (not further specified).	5
Meding^30^	2008	Sweden	Cross‐sectional	Occupational/general population (bakers, hairdressers, dental technicians, controls from general population)	1761^†^	13 452	Self‐reported	HE not specified	Yes/no + amount	Smoking was negatively associated with HE in bakers PPR 0.67, 95% CI 0.49‐0.92. No association was found for the other groups. Amount of smoking was positively associated with HE in hairdressers (smoking >10 cigarettes/d in hairdressers: 22.6% HE, <10 cigarettes/d: 17.4% HE (*P* = 0.01)).	7
Thyssen^31^	2009	Denmark	Cross‐sectional	General population	748	3471	Self‐reported	All types of HE further categorized as: Atopic HE/ allergic HE/ allergic and atopic HE /other HE	Never, former, current + amount	Smoking was positively associated with HE Previous: OR 1.13, 95% CI 0.90‐1.40, Current <15 g daily: OR 1.51, 95% CI 1.14‐2.02, Current >15 g daily: OR 1.38, 95% CI 0.99‐1.92.	7
Meding^32^	2010	Sweden	Cross‐sectional	General population	2344	25 428	Self‐reported	HE not specified	Never/former/current/ occasional (not daily) + amount	Former smoking and smoking >15 cigarettes/d was positively associated with HE in the last year Ex‐smokers: PPR 1.14, 95% CI 1.04‐1.25; Occasional smokers: PPR 1.01, 95% CI 0.84‐1.21, 1‐7 cigarettes/d: PPR 1.10, 95% CI 0.92‐1.32, 8‐15 cigarettes/d: PPR 1.18, 95% CI 1.00‐1.39, >15 cigarettes/d: PPR 1.40, 95% CI 1.15‐1.71) There was a dose–response relation between amount of cigarettes and HE. (PPR 1.05 (*P* < 0.001)).	8
Röhrl^33^	2010	Sweden	Cross‐sectional	General population (upper secondary school children)	350^†^	6095	Self‐reported	HE not specified	Yes/no	No association between smoking and HE (OR 0.85, 95% CI 0.59‐1.23)	7
Stenberg^34^	2010	Sweden	Cross‐sectional	General population	6135^†^	65 261	Self‐reported	HE not specified	Yes/no and daily snuff use	Daily smoking and the use of snuff were positively associated with HE (smoking: OR 1.18, 95% CI 1.09‐1.27; snuff use: OR 0.88, 95% CI 0.80‐0.97)	7
Anveden Berglind^35^	2011	Sweden	Cross‐sectional	General population	2746	27 793	Self‐reported	HE not specified	Yes/no	Smoking was positively associated with HE (PPR 1.025, 95%CI 1.006‐1.044).	7
Kütting^36^	2011	Germany	Prospective cohort	Occupational (metalworkers)	217	1020	Self‐reported/physician diagnosed	HE not specified	Yes/no	No association between smoking and HE.	4
Ibler^5^	2012	Denmark	Cross‐sectional	Occupational (healthcare workers)	397^†^	2269	Self‐reported	HE not specified	Not specified	No association between smoking and HE (not further specified).	3
Johannisson^37^	2013	Sweden	Prospective cohort	General population (upper secondary school children)	500	1516	Self‐reported	HE not specified	Yes/ no + amount	Persons with HE in 2008 smoked more cigarettes than persons without HE ever (*P* = 0.012) No association between amount of smoking and HE ever.	7
Mortz^38^	2014	Denmark	Prospective cohort	General population(school children)	127	891	Self‐reported and point prevalence physician‐diagnosed	HE not specified	Yes/no	No association between smoking and HE (OR 1.4, 95% CI 0.9‐2.1).	5
Patruno^39^	2014	Italy	Case‐control	General population (housewives)	214	516	Self‐reported	Chronic HE not specified	Yes/no + amount	No association between smoking and HE (*P* = 0.859).	5
Hougaard^40^	2014	Denmark	Cross‐sectional	Occupational/general population (hairdressers and controls from general population)	437	1904	Self‐reported	HE not specified	Never/former/current + amount	Smoking was not associated with HE (not further specified).	6
Wrangsjö^41^	2015	Sweden	Cross‐sectional	General population	2681	27 466	Self‐reported	HE not specified	Yes/no	Daily snuff use was negatively associated with HE and smoking was not associated with HE (PPR 0.813, 95% CI 0.686‐0.964; and PPR 1.023, 95% CI 0.848‐1.234, respectively).	7
Lai^42^	2016	USA	Cross‐sectional	General population	38	1301	Physician‐diagnosed	HE not specified	Non/current/smoked at least 100 cigarettes + amount (g/d)	Smoking was positively associated with HE. (current: OR 4.02, 95% CI 1.13‐14.24; >15/d: OR 4.69, 95% CI 1.17‐18.76; <15/d: OR 3.82, 95% CI 0.89‐16.36.; Smoked at least 100 cigarettes: OR 1.21, 95% CI 0.58‐2.52)	7
Vindenes^43^	2017	Norway	Cross‐sectional	General population	5757	50 781	Self‐reported	HE not specified	Never, former, current	Smoking was positively associated with HE. (former: RR 1.11, 95% CI 1.05‐1.19 ; current: RR 1.17, 95% CI 1.09‐1.26)	7
Van der Heiden^44^	2018	Denmark	Retrospective cohort	Clinical	120	120	Medical reports	Hyperkeratotic endogenous HE/ irritant contact dermatitis/ allergic contact dermatitis/ Atopic HE/ contact urticaria/ vesicular endogenous HE	Yes/no	Smoking was positively associated with HE (*P* = 0.049).	8
Hamnerius^45^	2018	Sweden	Cross‐sectional	Occupational (healthcare workers)	1870	9051	Self‐reported	HE not specified	Yes/no (daily)	No association between smoking and HE (OR 1.15, 95% CI 0.84‐1.57).	6
Hajaghazadeh^46^	2018	Iran	Case‐control	General population (housewives and hairdressers)	158	770	Self‐reported	HE not specified	Yes/no	Smoking was positively associated with HE (OR 3.44, 95% CI 1.73‐6.85).	5
Erdem^47^	2020	Turkey	Cross‐sectional	Occupational (health care workers)	54	107	Physician diagnosed	HE not specified	Yes/no	No association between smoking and HE.	3
Jing^48^	2020	China	Cross‐sectional	General population (adolescents)	674	20 129	Physician diagnosed	All types of HE further categorized as: Interdigital eczema/ recurrent vesicular HE/other types (combined chronic fissured HE, hyperkeratotic HE, nummular HE)	Yes/no	No association between active smoking and HE (OR 1.33, 95% CI 0.68‐2.60)	9
Chiriac^49^	2020	Romania	Cross‐sectional	Occupational (health care workers)	247	247	Self‐reported	HE not specified	Yes/no + amount and duration in years	No association between years of smoking and HE. No association between number of cigarettes/d and HE.	2
Falay Gür^50^	2021	Turkey	Cross‐sectional	Occupational (health care workers)	308	601	Self‐reported	HE not specified	Yes/no	No association between smoking and HE.	3
Incidence											
Lerbaek^51^	2007	Denmark	Prospective cohort	General population (twins)	244	3297	Self‐reported	HE not specified	Never, former, current +≤15/>15 pack years	No association for current or former smokers and HE (IRR: 1.1, 95% CI 0.8‐1.5; and 1.1, 95% CI 0.8‐1.6, respectively) No association for pack‐years and HE.	7
Reich^52^	2020	Germany	Prospective controlled intervention study	Occupational (metal work apprentices)	83	421	Physician diagnosed and self‐reported	HE not specified	Yes/no + amount	Smoking was positively associated with incident HE (*P* < 0.01). The amount of cigarettes smoked was not associated with incident HE.	8
Subtype of hand eczema											
Weigl^53^	2011	Germany	Case‐control	Clinical	132	132	Self‐reported	Dyshidrotic HE	Yes/no	No association between smoking and vesicular HE compared to non‐vesicular HE (OR 1.10, 95% CI 0.43‐2.81).	7
Molin^54^	2014	Germany	Cross‐sectional	Clinical	153	153	Physician diagnosed	All types of chronic HE further categorized as: Allergic contact dermatitis/combined allergic and irritant contact dermatitis/ atopic HE/ idiopathic HE/ dyshidrotic HE/ hyperkeratotic HE/ mixed HE	Never, former, current	Smoking was positively associated with combined allergic and irritant HE (*P* < 0.05) but not with other forms of HE.	6
Brans^55^	2016	Germany	Retrospective cohort	Occupational	723	723	Medical records	Atopic HE/irritant contact dermatitis/allergic contact dermatitis/ hyperkeratotic HE/ HE with erythema and desquamation/vesicular HE	Never/former/current + amount	Smoking was positively associated with vesicular HE and negatively associated with hyperkeratotic HE (both *P* < 0.001). In multivariate logistic regression analysis smoking was positively associated with vesicular HE (OR 2.86, 1.59–5.13). No association for the other subtypes of HE was found.	7
Van der Heiden^44^	2018	Denmark	Retrospective cohort	Clinical	120	120	Medical reports	Hyperkeratotic endogenous HE irritant contact dermatitis/ allergic contact dermatitis/ Atopic HE/ contact urticaria/ vesicular endogenous HE	Yes/no	Prevalence of hyperkeratotic HE and smoking compared to other subtypes of HE (OR 1.00, 95% CI 0.27‐3.74).	8
Brans^56^	2020	Germany	Prospective cohort	Clinical occupational	197	197	Physician diagnosed	All types of HE further categorized as: Atopic HE/ irritant contact dermatitis/ allergic contact dermatitis/ hyperkeratotic HE	Yes/no + amount	Smoking was positively associated with vesicular HE in subjects taking part in the tertiary individual prevention program (*P* < .05 at baseline and *P* < .001 at follow‐up) but not in the secondary prevention program. Smoking was not associated with any of the other subtypes of HE in both prevention programs.	4
Obermeyer^57^	2021	Germany	Retrospective cohort	Clinical occupational	1614	1614	Physician diagnosed	All types of HE further categorized as: Atopic HE/ irritant contact dermatitis/ allergic contact dermatitis/ hyperkeratotic HE/vesicular HE	Yes/no	Vesicular HE was more frequent among smokers than non‐smokers (45.7% vs 26.9%, *P* < .001).	4
**Severity**											
Ibler^5^	2012	Denmark	Cross‐sectional	Occupational (healthcare workers)	397	2269	Self‐reported	HE not specified	Not specified	No association between smoking and severity of HE (not further specified).	3
Brans^58^	2014	Germany	Prospective cohort	Occupational (healthcare, metal industry, hairdressing trade)	1608	1608	Physician diagnosed	All types of HE further categorized as: Atopic HE/ irritant contact dermatitis/ allergic contact dermatitis	Yes/no + amount	Smoking was positively associated with severity of HE (*P* < 0.01/*P* = 0.02). No dose‐dependent association between the amount of cigarettes and severity of HE was reported.	5
Patruno^39^	2014	Italy	Case‐control	General population (housewives)	214	516	Self‐reported	Chronic HE not specified	Yes/no + amount	Smoking amount was negatively associated with severity of HE. Almost clear HE was more frequent among smokers (*P* < 0.05). “Severe” form more frequently in non‐smokers (*P* = 0.01). No differences: mild and moderate disease (*P* = 0.39 and *P* = 0.17). No dose‐dependent association with amount of cigarettes was found.	5
Sørensen^59^	2017	Denmark	Cross‐sectional	Occupational	773	773	Self‐reported	HE not specified	Yes/no	Smoking was positively associated with severity of HE (OR 2.15, 95% CI 1.29‐3.59).	7
Hafsia^60^	2019	Tunisia	Cross‐sectional	Occupational (administrative, employee, labor, technician, doctor, paramedical staff)	109	109	Physician diagnosed	HE not specified	Never/former/current	No association between smoking and severity of HE.	5
Brans^56^	2020	Germany	Prospective cohort	Clinical occupational	197	197	Physician diagnosed	All types of HE further categorized as: Atopic HE/ irritant HE/ allergic contact dermatitis/ hyperkeratotic HE	Yes/no + amount	No association between smoking or smoking amount and severity of HE.	4
Falay Gür^50^	2021	Turkey	Cross‐sectional	Occupational (health care workers)	308	601	Self‐reported	HE not specified	Yes/no	No association between smoking and severity of HE.	3
Prognosis											
Douwes^61^	2000	Germany	Retrospective cohort	Clinical	62	62	Medical report	Palmoplantar eczema	Yes (>10 cigarettes)/no	Smoking was associated with a worse prognosis of HE (*P* < 0.05).	6
Veien^29^	2008	Denmark	Prospective cohort	Private dermatological practice	522	522	Physician diagnosed	HE not specified	Yes/no	No association between smoking and long‐standing HE (not further specified).	4
Olesen^62^	2019	Denmark	Retrospective cohort	Clinical occupational	1491	1491	Medical records	HE not specified	Never/former/current	Smoking was associated with persistence of HE (OR 0.48, 95% CI 0.31‐0.72).	6
Obermeyer^57^	2021	Germany	Retrospective cohort	Clinical occupational	1614	1614	Physician diagnosed	All types of HE further categorized as: Atopic HE/ irritant contact dermatitis/ allergic contact dermatitis/ hyperkeratotic HE/vesicular HE	Yes/no	Nearly 58.4% of tobacco smokers claimed no response or worsening of HE vs 47.6% of the non‐smokers (*P* = .134).	4
**Stress**											
**Prevalence**											
Anveden Berglind^35^	2011	Sweden	Cross‐sectional	General population	2747^†^	27 793	Self‐reported	HE not specified	How often do you feel stressed? High exposure: a couple of days per week/ most days of the week. Low exposure: Never or a few times per year/about once a month/about 1 d per week.	Stress was positively associated with HE in the past year (PPR 1.326, 95% CI 1.303‐1.350).	7
Magnavita^63^	2011	Italy	Cross‐sectional	Occupational (healthcare workers)	138	1744	Physician diagnosed	HE not specified	Occupational stress factors (demand/control/support model): Job control, job demands, social support, high strain, high iso‐strain.	High job demands, high strain, and high iso‐strain were positively associated with current HE (OR 1.13, 95% CI 1.06‐1.22; OR 1.91, 95% CI 1.29‐2.91; and OR 2.07, 95% CI 1.37‐3.11, respectively). High social support and higher job control were negatively associated with current HE (OR 0.87, 95% CI 0.82‐0.91; and OR 0.93, 95% 0.88‐0.98).	10
Wrangsjö^41^	2015	Sweden	Cross‐sectional	General population	2681	27 466	Self‐reported	HE not specified	How often do you feel stressed? High exposure: a couple of days per week/ most days of the week. Low exposure: Never or a few times per year/about once a month/about 1 day per week.	Stress was positively associated with HE in the past year (PPR 1.528, 95% CI 1.420‐1.643).	7
Marron^1^	2018	Europe	Case‐control	Clinical	143	1496	Physician diagnosed	HE not specified	Stressful life event in the last 6 mo (yes/no)	Reporting a stressful life event was associated with HE (*P* = 0.044 (f), *P* = 0.045 (m))	7
Hamnerius^45^	2017	Sweden	Cross‐sectional	Occupational (healthcare workers)	1870	9051	Self‐reported	HE not specified	How often do you feel stressed? Never or only a few times per year, sometime every month, sometime every week, some days every week, most days of the week	Stress was positively associated with HE in the past year. Some time every month: OR 1.1, 95% CI 0.9‐1.5; sometime every week: OR 1.4, 95% CI 1.1‐1.9; some days every week: OR 2.0, 95% CI 1.5‐2.6; most days of the week: OR 2.2, 95% CI 1.6‐3.1.	6
Falay Gür^50^	2021	Turkey	Cross‐sectional	Occupational (health care workers)	308	601	Self‐reported	HE not specified	Exposure to stress: Once a week or less vs more than once a week	No association between exposure to stress and HE.	4
**Severity**											
Lodi^64^	1992	Italy	Case–control study	Clinical	104	312	Physician diagnosed	Pompholyx	Emotional stress as aggravating factor (yes/no)	18/104 (17.3%) mentioned stress as aggravating factor of HE	2
Veien^29^	2008	Denmark	Prospective cohort	Clinical	522	522	Physician diagnosed	HE not specified	Psychological stress as aggravating factor (yes/no)	Men (6%) and women (13%) mentioned stress as aggravating factor.	5
Böhm^65^	2014	Germany	Cross‐sectional	Occupational	122	122	Physician diagnosed	HE not specified	High chronic stress or low chronic stress (based on the mean TICS‐score)	No association between chronic stress and severity of HE.	5
Sørensen^59^	2016	Denmark	Cross‐sectional	Occupational	773	773	Self‐reported	HE not specified	How often do you feel stressed? A few times a year, approximately once a month, weekly, a few times a week, most days.	No association between stress and current severity of HE.	8
Hafsia^60^	2019	Tunisia	Cross‐sectional	Clinical	109	109	Physician diagnosed	HE not specified	Score of PSS‐10 score ≤ 27 or > 27, and occupational stress Siegrist's “effort reward imbalance” questionnaire (ratio > 1 defines an imbalance between efforts and rewards).	No association between stress and severity of HE.	6
Janardhanan^66^	2020	India	Cross‐sectional	Clinical	62	62	Physician diagnosed	HE not specified	Emotional stress as aggravating factor	25/62 (40.3%) mentioned emotional stress as aggravating factor of HE.	3
Falay Gür^50^	2021	Turkey	Cross‐sectional	Occupational (health care workers)	308	601	Self‐reported	HE not specified	1. Stress as aggravating factor (yes/no) 2. Exposure to stress: Once a week or less vs more than once a week	1. 9/308 (2.9%) mentioned stress as aggravating factor of HE. 2. No association between exposure to stress and severity of HE.	4
**Prognosis**											
Olesen^62^	2019	Denmark	Retrospective cohort	Occupational	1491	1491	Medical records	HE not specified	How often do you feel stressed?Low: A few times per year, approximately Once per month. High: Weekly, a couple of times per week, most days.	Higher level of stress was associated with persistence of HE (OR 0.72, 95% CI 0.53‐0.97).	6
**BMI**											
Prevalence											
Anveden Berglind^35^	2011	Sweden	Cross‐sectional	General population	2747^†^	27 793	Self‐reported	HE not specified	>30 or ≤ 30	BMI >30 was positively associated with HE in the past year (PPR 1.204, (95%CI 1.174‐1.234)	7
Wrangsjö^41^	2015	Sweden	Cross‐sectional	General population	2681	27 466	Self‐reported	HE not specified	>30 or ≤ 30	BMI >30 was positively associated with HE in the past year (PPR 1.232, 95% CI 1.104‐1.376).	5
Lai^42^	2016	USA	Cross‐sectional	General population	38	1301	Physician diagnosed based on photographs	HE not specified	Continuous variable	No association between BMI and current HE.	5
Vindenes^43^	2017	Norway	Cross‐sectional	General population	5757	50 781	Self‐reported	HE not specified	<18.5, ≥18,5‐ <25, ≥25‐ < 30, ≥30	BMI >30 was positively associated with HE (RR 1.11, 95% CI 1.03‐1.20).	8
Hamnerius^45^	2017	Sweden	Cross‐sectional	Occupational (healthcare workers)	1870	9051	Self‐reported	HE not specified	<30 and ≥ 30	BMI was positively associated with HE (OR 1.35, 95% CI 1.03‐1.78).	5
Subtype of HE											
Van der Heiden^44^	2018	Denmark	Retrospective cohort	Clinical	120	120	Medical reports	Hyperkeratotic endogenous HE/ irritant contact dermatitis/ allergic contact dermatitis/ Atopic HE/ contact urticaria/ vesicular endogenous HE	≤24.9 and > 24.9	No association between BMI and hyperkeratotic HE compared to other subgroups of HE (OR 0.87, 95% CI 0.27‐2.78).	8
Cazzaniga^67^	2018	Switzerland and Germany	Cross‐sectional	Clinical	1466	1466	Physician‐diagnosed	Vesicular HE/ Hyperkeratotic HE fingertip dermatitis	<25.0, 25.0‐29.9 and ≥ 30.0	In the semantic map analysis there seemed to be a link between a BMI >30, fingertip dermatitis, and hyperkeratotic HE with additional involvement of the feet.	7
**Severity**											
Sørensen^59^	2016	Denmark	Cross‐sectional	Occupational	773	773	Self‐reported	HE not specified	≤24.9 and > 24.9	No association between BMI and severity of HE.	7
Cazzaniga^68^	2016	Switzerland	Cross‐sectional	Clinical	199	199	Physician‐diagnosed	Fingertip dermatitis/ hyperkeratotic HE/ vesicular HE	<25.0, 25.0‐29.9 and ≥ 30.0	No association between BMI and moderate to severe HE (BMI 25.0‐29.9: OR 1.09, 95% CI 0.52–2.27, and BMI ≥30.0: OR 1.12, 95% CI 0.42–2.96).	7
Cazzaniga^67^	2018	Switzerland and Germany	Cross‐sectional	Clinical	1466	1466	Physician‐diagnosed	Fingertip dermatitis/ hyperkeratotic HE/ irritant contact dermatitis/vesicular HE	<25.0, 25.0‐29.9 and ≥ 30.0	Association between BMI ≥30.0 and severe chronic HE (only reported for the cohort of Switzerland, n = 199).	7
Hafsia^60^	2019	Tunisia	Cross‐sectional	Clinical	109	109	Physician diagnosed	HE not specified	Normal vs. overweight and obesity (not further specified)	No association between BMI and severity of HE (OR 1.08, 95%CI 0.47–2.47).	4
**Prognosis**											
Olesen^62^	2019	Denmark	Retrospective cohort	Occupational	1491	1491	Medical records	HE not specified	<18.5, 18.5‐ < 25, 25‐ < 30 and ≥ 30	No association between BMI and persistence of HE (BMI <18.5: OR 0.26, 95%CI 0.03–2.01, BMI 18.5‐ <25 reference category, BMI 25‐ < 30: OR 0.89 95%CI 0.64–1.24, BMI ≥30: OR 1.01 95%CI 0.69–1.49)	6
Cazzaniga^69^	2018	Switzerland	Prospective cohort	Clinical	199	199	Diagnosed by physician	Vesicular HE/ Hyperkeratotic‐fissured HE/ allergic contact dermatitis/ fingertip dermatitis/ atopic HE/irritant contact dermatitis	<25.0, 25.0‐29.9, ≥30.0	No significant difference in PGA MCID 6 mo (BMI: 25.0‐29.9: OR 1.00, 95% CI 0.51‐1.95 and BMI: ≥30.0: OR 0.95, 95% CI 0.39‐2.31). No significant difference in PGA change up to 24 mo after baseline (BMI: 25.0‐29.9: 0.12, 95% CI −0.15 to 0.39 and BMI: ≥30.0: 0.05 95% CI −0.36 to 0.46).	8
**Physical activity**											
**Prevalence**											
Kavli^70^	1984	Norway	Cross‐sectional	General population	1322	14 667	Self‐reported	Allergic eczema of the hands	Sedentary work, work leading to much walking, work leading to much walking and lifting, heavy manual labor	With the exception of the small group doing heavy manual work, a trend toward increasing frequency of HE with greater physical activity was found in women (sedentary work: 11.5%, work leading to much walking: 13.6%, work leading to much walking and lifting: 15.8%, heavy manual labor: 6.9%); this trend was not seen in men (5.0%, 5.0%, 4.9%, and 4.2%, respectively).	5
Pitché^71^	2006	France	Case‐control	Clinical	100	300	Physician diagnosed	Pompholyx	Sport (yes/no)	Positive association between sport and HE (OR 8.8, 95% CI 3.9‐20.8). After adjustment no significant association.	6
Anveden Berglind^35^	2011	Sweden	Cross‐sectional	General population	2747^†^	27 793	Self‐reported	HE not specified	How much exercise and physical exertion have you engaged in during your leisure time over the past 12 mo? High: Regular exercise and workouts or moderate regular exercise during leisure time. Low: Sedentary leisure time, or moderate exercise during leisure time	Physical activity during leisure time was negatively associated with HE in the past year (PPR 0.818, 95% CI 0.804‐0.832).	7
Ibler^5^	2012	Denmark	Cross‐sectional	Occupational (healthcare workers)	397	2269	Self‐reported	HE not specified	Not specified	No association between physical activity and HE (not further specified).	3
Johannisson^37^	2013	Sweden	Prospective cohort	General population (upper secondary school children)	500^†^	1516	Self‐reported	HE not specified	Hours per week doing sports	No association between hours per week doing sports and HE (HE (mean, median, Q1‐Q3) 4.1, 2, (1 – 4); no HE: 4.1, 2, (1 – 4); *P* > 0.05.	8
Lai^72^	2015	USA	Cross‐sectional	General population	42	2688	Physician diagnosed based on photographs	HE not specified	Vigorous physical activity (>10 min), moderate physical activity (>10 min), average daily activity (sits during the day and does not walk about very much, stand or walk about a lot during the day, lift light load or has to climb stairs or hills often, heavy work or carries heavy loads, total time spent on walking/cycling, mean MET scores)	Moderate and vigorous physical activity was negatively associated with present HE (OR 0.515. *P* = 0.043 and OR 0.396. *P* = 0.011, respectively). Lifting heavy weights was positively associated with HE (OR 2.743. *P* = 0.075). Mean MET score and total time spent on walking/cycling were not associated with HE. Mean MET score daily activities was positively associated with HE (OR 1.088, *P* = 0.042).	7
Wrangsjö^41^	2015	Sweden	Cross‐sectional	General population	2681	27 466	Self‐reported	HE not specified	How much exercise and physical exertion have you engaged in during your leisure time over the past 12 mo? High: Regular exercise and workouts or moderate regular exercise during leisure time. Low: Sedentary leisure time, or moderate exercise during leisure time.	Physical activity was negatively associated with HE in the past year (PPR 0.780, 95% CI 0.724‐0.840).	5
Jing^48^	2020	China	Cross‐sectional	General population (adolescents)	674	20 129	Physician diagnosed	All types of HE further categorized as: Interdigital eczema/ recurrent vesicular HE/ other types (combined chronic fissured HE, hyperkeratotic HE, nummular HE)	Minutes per week no/1‐419 and ≥420 min/wk	No association between physical activity and HE (1‐419 min: OR 0.88, 95% CI 0.71‐1.09; ≥420 min; OR 0.99, 95% CI 0.82‐1.18).	8
**Subtype of HE**											
Van der Heiden^44^	2018	Denmark	Retrospective cohort	Clinical	120	120	Medical reports	Hyperkeratotic endogenous HE/ irritant contact dermatitis/ allergic contact dermatitis/ Atopic HE/ contact urticaria/ vesicular endogenous HE	Almost physically passive or only light physical activity for <2 h/wk, light physical activity for 2‐3 h/wk, light physical activity for >4 h/wk or more strenuous physical activity for >4 h/wk, or regular hard training or competitions several times per week.	No association between physical activity and hyperkeratotic HE compared to other subtypes (light physical activity for 2‐3 h/wk: OR 2.72, 95% CI 0.20‐36.5; light physical activity for >4 h/wk or more strenuous physical activity for 2‐4 h/wk: OR 2.46, 95% CI 0.20‐30.4; more strenuous physical activity for >4 h/wk or regular hard training or competitions several times/wk: OR 5.66, 95% CI 0.33‐97.6.	8
**Severity**											
Ibler^5^	2012	Denmark	Cross‐sectional	Occupational (healthcare workers)	397	2269	Self‐reported	HE not specified	Not specified	No association between physical activity and severity of HE (not further specified).	3
Hafsia^60^	2019	Tunisia	Cross‐sectional	Clinical	109	109	Physician diagnosed	HE not specified	Yes/no (sports and leisure time separately)	No association between sports and severity of HE (OR 0.48, 95% CI 0.13–1.70); leisure activity was negatively associated with HE (OR 0.27, 95% CI 0.09–0.80).	4
**Prognosis**											
Olesen^62^	2019	Denmark	Retrospective cohort	Occupational	1491	1491	Medical records	HE not specified	Physical activities during spare time, including transportation to and from work within the last year. Light ≤3 h weekly, light ≥4 h weekly or moderate 2‐4 h weekly, moderate >4 h weekly or regular strenuous exercise.	Moderate >4 h weekly or regular strenuous exercise was associated with less persistence of HE (OR 1.93, 95% CLI 1.16‐3.21).	6
**Alcohol consumption**											
**Prevalence**											
Smith^73^	2005	China	Cross‐sectional	Occupational (clinical nurses)	50	282	Self‐reported	HE not specified	Occasional alcohol consumption or no alcohol consumption	No association between alcohol consumption and HE (OR 12.9, 95% CI 0.03–11 681.2).	3
Bø^28^	2008	Norway	Cross‐sectional	General population	1096	18 747	Self‐reported	HE not specified	Alcohol intake: 4‐7 times/wk, 2‐3 times/wk, approximately once a week, 2‐3 times per month, approximately once a month, sometimes last year, not last year, never	Except for a reduced reporting of HE among women drinking 4–7 times no association between alcohol consumption and HE was found (M: 4–7 times/wk: OR 0.97, 95% CI 0.45‐2.09; 1‐3 times/wk: OR 1.35, 95% CI 0.71‐2.57; sometimes last year up to 2‐3 times/mo: OR 1.09, 95% CI 0.57‐2.11) (F: 4–7 times/wk: OR 0.37, 95% CI 0.20‐0.68; 1‐3 times/wk: OR 0.73, 95% CI 0.51‐1.05; sometimes last year up to 2‐3 times/mo: OR 0.83, 95% CI 0.59‐1.17)	6
Thyssen^31^	2009	Denmark	Cross‐sectional	General population	748	3471	Self‐reported	All types of HE further categorized as: Atopic HE/ allergic HE/ allergic and atopic HE /other HE	Amount: 0, 1‐7, 8‐14 and ≥ 15 drinks weekly	No association between alcohol consumption and HE (1‐7: 0.84, 95% CI 0.63‐1.11; 8‐14: 0.80, 95% CI 0.57‐1.12; ≥15: 0.82, 95% CI 0.58‐1.16).	7
Anveden Berglind^35^	2011	Sweden	Cross‐sectional	General population	2746	27 793	Self‐reported	HE not specified	>35 g/d in men and > 25 g/d in women / <35 or <25 g/d	Alcohol consumption was negatively associated with HE (PPR 0.978, 95% CI 0.961‐0.995).	7
Lai^42^	2016	USA	Cross‐sectional	General population	38	1301	Physician diagnosed based on photographs	HE not specified	At least 12 drinks in the past year (yes/no)	No association between alcohol consumption and HE (OR 0.86, 95% CI 0.37‐1.97).	6
Jing^48^	2020	China	Cross‐sectional	General population (adolescents)	674	20 129	Physician diagnosed	All types of HE further categorized as: Interdigital eczema/ recurrent vesicular HE/ other types (combined chronic fissured HE, hyperkeratotic HE, nummular HE)	Yes/no	No association between alcohol consumption and HE (OR 0.96, 95% CI 0.64‐1.43).	8
**Incidence**											
Lerbaek^51^	2007	Denmark	Prospective cohort	General population (twins)	244	3297	Self‐reported	HE not specified	Never, ≤21, >21 drinks/wk	No association between alcohol consumption and incidence of HE (≤ 21 drinks: IR 1.3, 95% CI 0.9‐1.8; >21 drinks: IR 1.05, 95% CI 0.7‐3.3).	7
**Severity**											
Hafsia^60^	2019	Tunisia	Cross‐sectional	Clinical	109	109	Physician diagnosed	HE not specified	Alcoholism yes/no (not further specified)	No association between alcohol consumption and severity of HE (OR 2.34, 95% CI 0.48–11.36; after adjustment no significant association).	4
**Diet**											
**Prevalence**											
Röhrl^33^	2010	Sweden	Cross‐sectional	General population (upper secondary school children)	350^†^	6095	Self‐reported	HE not specified	Vegetarianism/veganism: Yes/no	No association between vegetarianism/veganism and HE in the past year (OR 0.97, 95% CI 0.67‐1.41).	7
Severity											
Counter^74^	1954	USA	Prospective cohort	Clinical	40	40	Physician diagnosed	Discoid eczema of de hands	Elimination diet excluding: Nuts, onions, tomatoes, chocolate, coffee, and pork. After clinical improvement: Food groups were restored to the diet one at a time and if no reaction after five days occurred the food group was considered safe for inclusion as a usual part of the diet.	Several patients had recurrences of vesicles within 5 days after introduction of particular food groups. Nuts (2), onions (5), tomatoes (1), chocolate (10), coffee (4), pork (2).	4
**Prognosis**											
Veien^75^	1983	Denmark	Prospective cohort	Clinical	202	202	Physician‐diagnosed	Vesicular HE	When patients reacted to the mixture as well as to one or two of the individual metal salts (nickel, cobalt or chromate), they were asked to reduce their daily oral intake of foods suspected of containing significant amounts of the metal(s).	Fifty‐six patients followed the diet. Thirty‐six patients cleared or improved after 1 mo of dieting. Twenty‐eight of them followed the prescribed diet rigorously or intermittently for at least a year, because they experienced recurrence of the dermatitis if they stopped. Six noted no long‐term benefit and two were lost to follow‐up.	3

*Note*: Studies occurring multiple times due to multiple studied lifestyle factors or outcome measures. When studies reported both unadjusted and adjusted results, adjusted results are reported. Cohort and case–control study outcomes with ≥6 points on the NOS, and cross‐sectional study outcomes with ≥7 points, were considered low risk of bias.

Abbreviations: n, number; HE, hand eczema; NOS, Newcastle‐Ottawa Scale, OR, odds ratio; CI, confidence interval; m, males; f, females; g, gram; PPR, population prevalence ratio; USA, United States of America; IR, incidence rate; RR, risk ratio; IRR, incidence rate ratio; TICS, Trier Inventory for the Assessment of Chronic Stress; PSS, Perceived Stress Scale; BMI; body mass index; PGA, Physician Global Assessment; MCID, minimal clinical important difference; MET, metabolic equivalent of task.

^†^ Calculated from reported percentages.

### 3.3 Smoking

**FIGURE 2:**
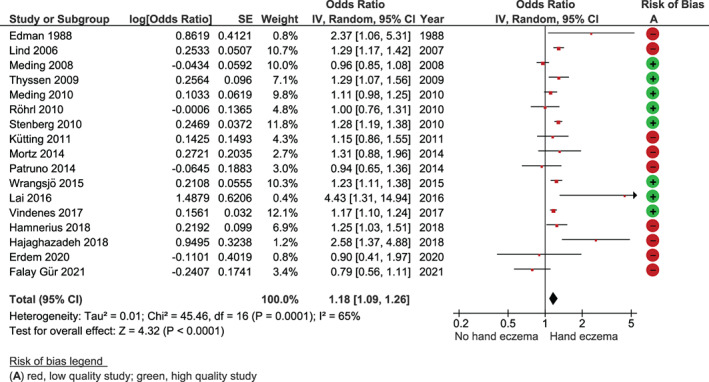
Forest plot smoking and prevalence of hand eczema. Cross‐sectional study outcomes with ≥7 points on the Newcastle‐Ottawa Scale were considered low risk of bias. Abbreviations: CI, confidence interval; df, degrees of freedom

Seventeen^23^, ^27^, ^30^, ^31^, ^32^, ^33^, ^34^, ^36^, ^38^, ^39^, ^41^, ^42^, ^43^, ^45^, ^46^, ^47^, ^50^ of the 29 included studies reporting the association between smoking and prevalence of HE could be included in a meta‐analysis. This included 23 983 subjects with HE and 191 352 subjects without HE, and showed a positive overall effect in the direction of HE compared with no HE (OR 1.18, 95% CI 1.09‐1.26) (Figure 2). Sub‐analysis of studies with a low risk of bias (N = 8)^30^, ^31^, ^32^, ^33^, ^34^, ^41^, ^42^, ^43^ demonstrated only a minor difference (OR 1.16, 95% CI 1.07‐1.26) (Figure S4). A sub‐analysis comparing studies in a non‐occupational setting (n = 11)^23^, ^31^, ^32^, ^33^, ^34^, ^38^, ^39^, ^41^, ^42^, ^43^, ^46^ versus an occupational setting (n = 6)^27^, ^30^, ^36^, ^45^, ^47^, ^50^ showed ORs of 1.21 (95% CI 1.12‐1.31) and 1.09 (95% CI 0.92‐1.28), respectively (Figures S5,S6). According to the GRADE approach, the quality of the evidence was very low (Table 2). Twelve studies could not be included in the meta‐analysis due to lack of data (n = 8),^5^, ^28^, ^29^, ^37^, ^40^, ^44^, ^48^, ^49^ categorization of smoking habits other than yes/no (n = 3),^24^, ^25^, ^26^ or outcome measures not convertible to ORs (n = 1).^35^ Of these, four reported a positive association between smoking and prevalence of HE,^24^, ^26^, ^35^, ^44^ and eight reported no association between smoking and HE.^5^, ^25^, ^28^, ^29^, ^37^, ^40^, ^48^, ^49^ Two studies on smoking and HE incidence found no association.^51^, ^52^ Regarding the association between smoking and specific subtypes of HE, no consistent results were found.^44^, ^53^, ^54^, ^55^, ^56^, ^57^ Of the seven studies reporting on smoking and severity of HE, results were contradictory, two studies reporting a positive association,^58^, ^59^ one a negative association,^39^ and four no association.^5^, ^50^, ^56^, ^60^ Two studies reported a worse prognosis for patients who smoked,^61^, ^62^ and two studies reported no association between smoking and longstanding HE or persistence of HE.^29^, ^57^ A possible publication bias was detected for the outcome smoking and prevalence of HE, based on asymmetric funnel plots (Figures S7 and S8). In combination with the findings above, this warrants caution regarding generalization.

**TABLE 2:** Summary of findings' table for the outcome smoking and prevalence of hand eczema

Quality assessment								Relative effect	
Outcome	Number of studies (participants)	Study design	Risk of bias	Inconsistency	Indirectness	Imprecision	Publication bias	OR (95% CI)	Quality of the evidence (GRADE)
Prevalence of hand eczema^a^	17 (215335)	Observational	Not serious	Serious^b^	Not serious	Not serious	Not serious	1.18 (1.09‐1.26)	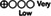

*Note*: The Quality of evidence was accessed using the GRADE approach. Explanations: GRADE Working Group grades of evidence. High quality: we are very confident that the true effect lies close to that of the estimate of the effect. Moderate quality: We are moderately confident in the effect estimate: The true effect is likely to be close to the estimate of the effect, but there is a possibility that it is markedly different. Low quality: Our confidence in the effect estimate is limited: The true effect may be markedly different from the estimate of the effect. Very low quality: Very little confidence in the effect estimate: The true effect is likely to be markedly different from the estimate of effect. Observational studies, such as the ones included in this systematic review, are assumed to have low quality and can be up‐ or downgraded based on the GRADE criteria.

Abbreviations: CI, confidence interval; OR, odds ratio.

^a^ Prevalence was either clinical diagnosed or self‐reported.

^b^ Downgraded for serious inconsistency: high heterogeneity. Clinically, observed in studies with participants from different settings (occupational, clinical, or general population) and outcomes (clinically confirmed or self‐report); Statistical heterogeneity observed as studies with inconsistent point estimates and low extent of 95% CI overlap with the meta‐analysis calculation.

### 3.4 Stress

Twelve studies reported the association between stress and HE on fourteen outcomes, of which seven^1^, ^35^, ^41^, ^45^, ^59^, ^62^, ^63^ outcomes had an overall low risk of bias. Stress was assessed in different ways. Five studies analyzing stress and prevalence of HE found a positive association^1^, ^35^, ^41^, ^45^, ^63^ and one reported no association.^50^ In four studies stress was mentioned as an aggravating factor,^29^, ^50^, ^64^, ^66^ and four studies did not find an association between stress and severity of HE.^50^, ^59^, ^60^, ^65^ One retrospective cohort study reported more persistence of occupational HE in subjects with higher self‐reported frequencies of stress.^62^

### 3.5 BMI

Twelve studies reported results on 13 outcomes of body mass index (BMI) and HE. Eight outcomes proved to have a low risk of bias.^35^, ^43^, ^44^, ^59^, ^67^, ^68^, ^69^ Five studies reported results on BMI and prevalence of HE, of which four reported a positive association between BMI ≥30 and HE.^35^, ^41^, ^43^, ^45^ One study found no association between BMI and prevalence of HE.^42^ Two studies included results on BMI and subtype of HE, with different results.^44^, ^67^ Three of four cross‐sectional studies reported no association between BMI and severity of HE,^59^, ^60^, ^68^ and one found a positive association between BMI and severe chronic HE in a subgroup of the included study population.^67^ One study did not find an association between BMI and the persistence of HE,^62^ and another study reported no significant difference in Physician Global Assessment (PGA) change for HE up to 24 months after baseline when comparing different categories of BMI.^69^

### 3.6 Physical activity

Eleven studies reported results on 12 outcomes of physical activity and HE, of which seven^35^, ^37^, ^42^, ^44^, ^48^, ^62^, ^71^ outcomes proved to have a low risk of bias. Eight studies included results on physical activity and prevalence of HE, of which four^5^, ^37^, ^48^, ^71^ found no association between physical activity and HE. One reported increasing HE frequencies with higher physical activity levels at work only in women.^70^ Two reported a negative association between physical activity during leisure time and HE.^35^, ^41^ Another study reported varying outcomes in multiple categories of physical activity with various associations with HE.^42^ One study found no association between physical activity and hyperkeratotic HE compared to other subtypes of HE.^44^ Two other studies found no association between physical activity and severity of HE.^5^, ^60^ One clinical occupational retrospective cohort study found that moderate physical activity >4 hours weekly or regular strenuous exercise was associated with less‐persistent HE.^62^

### 3.7 Alcohol consumption

Seven included studies reported on the association between alcohol consumption and HE, of which four^31^, ^35^, ^48^, ^51^ of the outcomes were assessed as having a low risk of bias. These studies used varying definitions of alcohol consumption. Six of them reported results on HE prevalence, of which five^28^, ^31^, ^42^, ^48^, ^73^ did not find an association between alcohol consumption and the prevalence of HE. One study reported a negative association between alcohol consumption and HE.^35^ No associations between alcohol consumption and incidence or severity of HE were reported.^51^, ^60^

### 3.8 Diet

Three studies reported an association between diet and HE, of which one study^33^ was of low risk of bias. That study did not find an association between a vegetarian/vegan diet and the prevalence of HE in upper‐secondary school children.^33^ Another study reported that all patients with vesicular HE who completed an elimination diet (n = 20/40) had recurrences of vesicles within 5 days after introducing particular food groups.^74^ The third study reported results of an elimination diet in 56 patients with vesicular (patch‐test negative) HE, who reacted to metal salts orally.^75^ Thirty‐six of the patients improved or cleared after 1 month of dieting and 28 of them followed the prescribed diet for at least 1 year because of recurrences when stopping the diet.

### 3.9 Sleep

No studies could be identified reporting the association between the amount of sleep and HE.

## 4 DISCUSSION

### 4.1 Main findings

The results of the meta‐analysis provided very low quality evidence that smoking is associated with a higher prevalence of HE. No firm conclusions could be drawn about the association between smoking and incidence, subtype, severity, or prognosis of HE. The limited available evidence may suggest a positive association between stress, BMI, and prevalence of HE, and a possible role for stress as an aggravating factor of HE. None of the studies reported a positive association between alcohol consumption and HE, or BMI, stress, physical activity, and severity of HE. Both positive and negative associations between physical activity and prevalence of HE were found. Data were insufficient to make a statement regarding the association between diet and HE and lifestyle factors and prognosis of HE in general. In addition, limited and contradictory evidence on lifestyle factors and the subtypes of HE was found.

### 4.2 Interpretation

Two previous studies, one meta‐analysis and a systematic review, also reported on smoking and HE.^76^, ^77^ No association between smoking and the prevalence of HE was found in the meta‐analysis. However, this conclusion was based on only three studies conducted in the same country.^76^ On the other hand, the systematic review indicated that smoking might cause an increased prevalence and severity of HE, especially in high‐risk occupations.^77^ The exact mechanism behind the association between lifestyle factors and HE remains unknown. It has been described that lifestyle factors such as smoking^11^ and stress^12^ may influence the immune system toward Th2 immunity. In addition, obesity is associated with a chronic low‐grade inflammatory state, which might also influence HE.^13^ Furthermore, it is also possible that a healthier lifestyle improves overall health, of which HE might benefit in conjunction. Although, the reverse might also defensible: HE impairs quality of life, which may negatively influence health behavior in subjects affected. However, as it is (almost) impossible to study lifestyle factors in designs other than observational studies, results should be interpreted with caution, as no conclusions on the direction of found associations can be drawn. Negative associations were reported for physical activity and HE. The direction of the association between physical activity might vary by the used definition of physical activity. It might be hypothesized that performing physical activity at work might contribute to the occurrence, severity, and prognosis of HE due to the exposure of irritants. On the other hand, physical activity during leisure time might be avoided by those affected by HE due to symptoms. For the majority of the lifestyle factors and outcomes of HE no convincing evidence was found. Yet this does not exclude that they might influence HE. The simplest explanation may be that the well‐known endogenous and exogenous factors are of far greater influence than lifestyle on the prevalence, severity, or prognosis of HE.

### 4.3 Heterogeneity and confounders

In the current systematic review, inconsistencies for all lifestyle factors and almost all outcomes were reported. Part of the conflicting results might be attributed to the high heterogeneity between studies, with varying study populations (clinical, occupational, and general population); with various levels of disease severity, underlying etiologies, concomitant of lifestyle factors, differences in study design; and the use of different, mostly not validated, measurement instruments to assess outcomes. The results of the sub‐analysis of smoking and prevalence of occupational HE were not in line with the systematic review published previously that reported an increased prevalence of HE, especially in high‐risk occupations.^77^ However, it should be noted that the current meta‐analysis was based on six studies, of which five had an overall high risk of bias. And a possible explanation might be that the overall contribution of smoking to the occurrence of HE is rather small and, especially in occupational settings with other more predominant risk factors such as exposure to allergens and irritants, the effect of smoking on HE might be of less significance than other risk factors.

In addition, confounding factors have to be considered, as both HE and lifestyle factors can be influenced by patient characteristics and environment, including age, sex, socioeconomic, and psychosocial factors. This is partly covered in the quality assessment, where comparability was scored based on adjustment for age and sex, resulting in a lower score for studies showing only unadjusted results. Multivariate analyses were reported with different combinations of included confounders, so data could not be pooled.

### 4.4 Strengths and limitations

This thorough and robust systematic review with meta‐analysis gives a comprehensive overview of the existing literature on a possible association between HE and multiple lifestyle factors. In addition, it includes, besides the occurrence of HE, several other clinically important outcomes as severity and prognosis of HE. Broad inclusion criteria were applied, with no restrictions on publication year, language, or study design. In accordance with the study design, the recommended instrument for quality assessment was chosen: the (adapted) Newcastle‐Ottawa Scale (or NOS). Moreover, the confidence in estimate of the pooled evidence for smoking and prevalence of HE was assessed with GRADE. However, there are also several limitations: because studies of lifestyle factors cannot be randomized, all evidence came from observational studies with high heterogeneity. The study setting, population, and design, together with the degree of adjustment for confounders, varied widely across the studies.

Due to the limited number of studies for each outcome, it was not possible to incorporate all these aspects into subgroup analyses. Heterogeneity did not permit, besides for smoking, meta‐analyses and assessment of pooled outcomes of major concern, and use GRADE for rating of the evidence. In addition, visual inspection of the funnel plots detected a possible risk of publication bias for smoking and HE. Furthermore, reporting bias could not be excluded. The majority of the studies, which could not be included in the meta‐analysis, due to lack of numerical data, reported no association between smoking and the occurrence of HE. If data were reported sufficiently it is not unimaginable that including those (in some cases large) studies would alter the overall estimated effect. Finally, information bias should also be taken into account, as self‐reported lifestyle behavior might be prone to influence by socially desirable answers.

This extensive systematic review and meta‐analysis found very low‐quality evidence that smoking is associated with the prevalence of HE. No convincing evidence of associations for the other lifestyle factors with HE were found. The data from this systematic review did not enable differentiation in setting, diagnosis, or population. Therefore, it is still possible that lifestyle factors might be more relevant for specific subgroups of patients with HE. This should be incorporated in the design of future studies. And if done so, these studies require the use of validated and well‐defined measurement instruments and need to be complete in reporting interpretational data.

## CONFLICT OF INTEREST

Dr Marie L.A. Schuttelaar is an advisor, consultant, speaker, and/or investigator for AbbVie, Pfizer, LEO Pharma, Regeneron, Sanofi Genzyme, Eli Lilly, and Galderma. She has received grants from Regeneron, Sanofi Genzyme, Novartis, and Pfizer.

## AUTHOR CONTRIBUTIONS

**Laura Loman:** Conceptualization (lead); data curation (lead); formal analysis (lead); investigation (lead); methodology (equal); project administration (lead); validation (equal); visualization (lead); writing – original draft (lead); writing – review & editing (supporting). **Marjolein J. Brands:** Conceptualization (supporting); investigation (equal); methodology (equal); writing – review and editing (supporting). **Anna A. L. Massella Patsea:** Investigation (equal); writing – review and editing (supporting). **Klaziena Politiek:** Supervision (supporting); writing – review and editing (supporting). **Bernd W. M. Arents:** Conceptualization (supporting); investigation (supporting); methodology (supporting); supervision (supporting); validation (equal); writing – review and editing ( (lead)). **Marie L. A. Schuttelaar:** Conceptualization (supporting); methodology (supporting); supervision (lead); validation (equal); writing – review & editing (lead).

## Supporting information


**Appendix S1** Supplementary Information

## Data Availability

Data available on request from the authors.
