# Supplementary material for: Lifestyle factors and hand eczema: A systematic review and meta‐analysis of observational studies
Source: Contact Dermatitis. 2022 Apr 2;87(3):211–32. doi: 10.1111/cod.14102 (PMC9541324; doi:10.1111/cod.14102)
Supplement: Supplementary file 1 — Appendix S1 Supplementary Information [file COD-87-211-s001.docx]

**Lifestyle factors and hand eczema: a systematic review and meta-analysis of observational studies**

L. Loman^1^, M.J. Brands^1^, A.A.L. Massella Patsea^1^, K. Politiek^3^, B.W.M. Arents^2^, M.L.A. Schuttelaar^1^

Names of the institutions:

1. University of Groningen, University Medical Center Groningen, Department of Dermatology, Groningen, the Netherlands
2. Dutch Association of People with Atopic Dermatitis, Nijkerk, the Netherlands
3. Medical Center Leeuwarden, Department of Dermatology, Leeuwarden, the Netherlands

Correspondence to: M.L.A. Schuttelaar, Department of Dermatology, University Medical Center Groningen, P.O. Box 30.001, 9700 RB Groningen, the Netherlands, phone +31503612520, mail: m.l.a.schuttelaar@umcg.nl, fax: +31503612624

**Online-only Supplements**

Appendix S1: Full search strategy for Pubmed, EMBASE, and Web of Science. 2

Table S2: List of excluded studies with justification. 3

Figure S3: Risk of bias graphs and summaries. 5

Figure S4: Forest plot smoking and prevalence of hand eczema, sub-analysis only including study outcomes with a risk of bias score ≥ 7. 20

Figure S5: Forest plot smoking and occurrence of hand eczema, only including study outcomes in a non-occupational setting. 20

Figure S6: Forest plot smoking and occurrence of hand eczema, subanalysis only including study outcomes in an occupational setting. 21

Figure S7: Funnel plot smoking and prevalence of hand eczema. Including all studies from the meta-analysis. 21

Figure S8: Funnel plot smoking and prevalence of hand eczema. Including all studies from the sub-analysis of study outcomes with a risk of bias score ≥ 7. 22

References of excluded studies 23

**Appendix S1: Full search strategy for Pubmed, EMBASE, and Web of Science.**

**Pubmed**

(("Hand"[Mesh] *AND* "Dermatitis"[Mesh]) OR ((”hand”[tiab] OR “hands”[tiab]) *AND* (“dermatitis”[tiab] *OR* “dermatos*”[tiab] *OR* “eczema*”[tiab])) OR "Hand Dermatoses"[Mesh] OR "Eczema, Dyshidrotic"[Mesh] OR ((“dyshidrotic*”[tiab] *OR* “vesicular*”[tiab] *OR* “palmoplantar*”[tiab]) *AND* “eczema*”[tiab]) OR "Acrodermatitis"[Mesh] OR “acrodermatit*”[tiab] OR “pompholyx”[tiab])

AND

("Life Style"[Mesh] OR “life style*”[tiab] OR “lifestyle*”[tiab] OR "Risk Factors"[Mesh] OR “risk*”[tiab] OR "Smoking"[Mesh] OR “smok*”[tiab] OR "Drinking Behavior"[Mesh] OR “drinking*”[tiab] OR “alcohol*”[tiab] OR "Exercise"[Mesh] OR “exercis*”[tiab] OR "Sports"[Mesh] OR “sport*”[tiab] OR “physical activ*”[tiab] OR “sedentar*”[tiab] OR “physical inactiv*”[tiab] OR "Body Weight"[Mesh] OR “body weight*”[tiab] OR "Body Mass Index"[Mesh] OR “Body Mass*”[tiab] OR “BMI”[tiab] OR “lean*”[tiab] OR “underweight*”[tiab] OR “obes*”[tiab] OR “overweight*”[tiab] OR "Stress, Psychological"[Mesh] OR “stress*”[tiab] OR "Sleep"[Mesh] OR “sleep*”[tiab] OR "Nutritional Sciences"[Mesh] OR “nutrition*”[tiab] OR "Diet, Food, and Nutrition"[Mesh] OR “diet*”[tiab])

2,302 results (20211014)

**EMBASE**
(('hand'/exp AND 'dermatitis'/exp) OR ((‘hand’:ti,ab,kw OR ‘hands’:ti,ab,kw) *AND* (‘dermatitis’:ti,ab,kw *OR* ‘dermatos*’:ti,ab,kw *OR* ‘eczema*’:ti,ab,kw)) OR 'hand eczema'/exp OR ‘hand dermatitis’/exp OR 'pompholyx'/exp OR ‘pompholyx’:ti,ab,kw OR ((‘dyshidrotic*’:ti,ab,kw *OR* ‘vesicular*’:ti,ab,kw *OR* ‘palmoplantar*’:ti,ab,kw) *AND* ‘eczema*’:ti,ab,kw) OR 'acrodermatitis'/exp OR ‘acrodermatit*’:ti,ab,kw)

***AND***

('lifestyle'/exp OR ‘life style*’:ti,ab,kw OR ‘lifestyle*’:ti,ab,kw OR 'risk factor'/exp OR ‘risk*’:ti,ab,kw OR 'smoking'/exp OR ‘smok*’:ti,ab,kw OR ‘alcohol*’:ti,ab,kw OR ‘drinking*’:ti,ab,kw OR 'physical activity, capacity and performance'/exp OR 'sport'/exp OR ‘exercis*’:ti,ab,kw OR 'inactivity'/exp OR ‘physical activ*’:ti,ab,kw OR ‘sedentar*’:ti,ab,kw OR ‘physical inactiv*’:ti,ab,kw OR ‘sport*’:ti,ab,kw OR 'body weight'/exp OR 'body mass'/exp OR 'leanness'/exp OR 'underweight'/exp OR 'obesity'/exp OR ‘body weight*’:ti,ab,kw OR ‘lean*’:ti,ab,kw OR ‘underweight*’:ti,ab,kw OR ‘BMI’:ti,ab,kw OR ‘obes*’:ti,ab,kw OR ‘overweight*’:ti,ab,kw OR ‘body mass*’:ti,ab,kw OR 'stress'/exp OR ‘stress*’:ti,ab,kw OR 'sleep'/exp OR ‘sleep*’:ti,ab,kw OR 'nutritional science'/exp OR 'nutrition'/exp OR ‘nutrition*’:ti,ab,kw OR ‘diet*’:ti,ab,kw OR ‘food*’:ti,ab,kw)

3,848 results (20211014)

**Web of Science**

(((“hand” OR “hands”) *AND* (“dermat*” *OR* “eczema*”)) OR ((“dyshidrotic*” *OR* “vesicular*” *OR* “palmoplantar*”) *AND* “eczema*”) OR “acrodermatit*” OR “pompholyx”)

**AND**(“life style*” OR “lifestyle*” OR “risk*” OR “smok*” OR “drinking*” OR “alcohol*” OR “exercis*” OR “sport*” OR “physical activit*” OR “sedentar*” OR “physical inactiv*” OR “body weight*” OR “body mass*" OR “BMI” OR “lean*” OR “underweight*” OR “obes*” OR “overweight*” OR “stress*” OR “sleep*” OR “nutrition*” OR “diet*” OR “food*”)

2.293 results (20211014)

**Table S2: List of excluded studies with justification.**

| **First author** | **Year** | **Reason of exclusion** |
| --- | --- | --- |
| Agner^1^ | 2009 | No lifestyle factor |
| Agner^2^ | 2000 | Review |
| Ahmed^3^ | 2015 | No lifestyle factor |
| Ahmed^4^ | 2021 | No hand eczema |
| Ali^5^ | 2020 | No lifestyle factor |
| Alluhayyan^6^ | 2020 | No lifestyle factor |
| Alsaidan^7^ | 2020 | No lifestyle factor |
| Apfelbacher^8^ | 2013 | Insufficient data |
| Apfelbacher^9^ | 2013 | No lifestyle factor |
| Arrandale^10^ | 2014 | No separate data hand eczema |
| Attwa^11^ | 2009 | No separate data hand eczema |
| Aydin^12^ | 2021 | No lifestyle factor |
| Babić^13^ | 2020 | No lifestyle factor |
| Becker^14^ | 1995 | Review |
| Bewley^15^ | 2013 | No lifestyle factor |
| Bjelajac^16^ | 2020 | Insufficient data |
| Boehm^17^ | 2012 | No lifestyle factor |
| Brans^18^ | 2015 | Review |
| Brans^19^ | 2015 | No separate data hand eczema |
| Celik^20^ | 2020 | No lifestyle factor |
| Chen^21^ | 2008 | Insufficient data |
| Chen^22^ | 2017 | No separate data hand eczema |
| Cheon^23^ | 2016 | No hand eczema |
| Chiriac^24^ | 2020 | No lifestyle factor |
| Chirico^25^ | 2004 | Insufficient data |
| Chou^26^ | 2016 | No hand eczema |
| Collier^27^ | 1956 | Case-report |
| Cvetkovski^28^ | 2006 | No lifestyle factor |
| Dalgard^29^ | 2015 | No lifestyle factor |
| Devold^30^ | 2019 | No lifestyle factor |
| Dietz^31^ | 2021 | No lifestyle factor |
| Dogan^32^ | 2021 | Report could not be retrieved |
| Dotterud^33^ | 2007 | No lifestyle factor |
| Drewitz^34^ | 2021 | No lifestyle factor |
| Fei^35^ | 2019 | No hand eczema |
| Fors^36^ | 2012 | No hand eczema |
| Gallicchio^37^ | 2011 | No hand eczema |
| Grabenhenrich^38^ | 2011 | No separate data hand eczema |
| Holm^39^ | 2016 | No separate data hand eczema |
| Hougaard^40^ | 2014 | No lifestyle factor |
| Huang^41^ | 2020 | No lifestyle factor |
| Kashyap^42^ | 2021 | No lifestyle factor |
| Kendziora^43^ | 2020 | No lifestyle factor |
| Khalili^44^ | 1999 | No separate data hand eczema |
| Lee^45^ | 2011 | No hand eczema |
| Liang^46^ | 2021 | No lifestyle factor |
| Linnainmaa^47^ | 1997 | No separate data hand eczema |
| Lysdal^48^ | 2014 | No hand eczema |
| Maarouf^49^ | 2018 | No separate data hand eczema |
| McArthur^50^ | 1992 | No hand eczema |
| Metin^51^ | 2020 | No lifestyle factor |
| Moldovan^52^ | 2021 | No lifestyle factor |
| Mortz^53^ | 2014 | No lifestyle factor |
| Murota^54^ | 2012 | Insufficient data |
| Nakamoto^55^ | 2018 | No hand eczema |
| Niemeier^56^ | 2002 | No separate data hand eczema |
| Nørreslet^57^ | 2018 | No lifestyle factor |
| O’neill^58^ | 2021 | No lifestyle factor |
| Patruno^59^ | 2020 | Review |
| Pourani^60^ | 2021 | No lifestyle factor |
| Pourani^61^ | 2021 | No lifestyle factor |
| Riala^62^ | 1998 | No lifestyle factor |
| Rizzi^63^ | 2021 | No lifestyle factor |
| Rönmark^64^ | 2012 | No separate data hand eczema |
| Rosén^65^ | 1988 | No lifestyle factor |
| Rystedt^66^ | 1989 | Review |
| Saha^67^ | 2021 | No lifestyle factor |
| Sato^68^ | 2004 | No hand eczema |
| Silverberg^69^ | 2015 | No hand eczema |
| Silverberg^70^ | 2018 | No separate data hand eczema |
| Simonsen^71^ | 2021 | No lifestyle factor |
| Singh^72^ | 2021 | No lifestyle factor |
| Singh^73^ | 2020 | No lifestyle factor |
| Smith^74^ | 2003 | No separate data hand eczema |
| Smith^75^ | 2005 | No separate data hand eczema |
| Smith^76^ | 2006 | No separate data hand eczema |
| Son^77^ | 2017 | No hand eczema |
| Svensson^78^ | 2013 | Review |
| Szepietowski ^79^ | 2002 | No lifestyle factor |
| Techasation^80^ | 2021 | No lifestyle factor |
| Thuesen^81^ | 2014 | Review |
| Veien^82^ | 1983 | No separate data hand eczema |
| Veien^83^ | 1987 | No separate data hand eczema |
| Veien^84^ | 1987 | No separate data hand eczema |
| Veien^85^ | 1983 | No separate data hand eczema |
| Veien^86^ | 1987 | <10 cases |
| Von Manteuffel^87^ | 2005 | Review |
| Weistenhöfer^88^ | 2019 | No separate data hand eczema |
| Xerfan^89^ | 2021 | Review |
| Yuan^90^ | 2021 | No hand eczema |
| Zeerak^91^ | 2021 | No lifestyle factor |

**Figure S3: Risk of bias graphs and summaries.**

***Smoking***

Risk of bias graph 1: review authors' judgements about each risk of bias item presented as percentages across all included cross-sectional study outcomes for smoking and hand eczema (prevalence, subtype and severity, n=42).


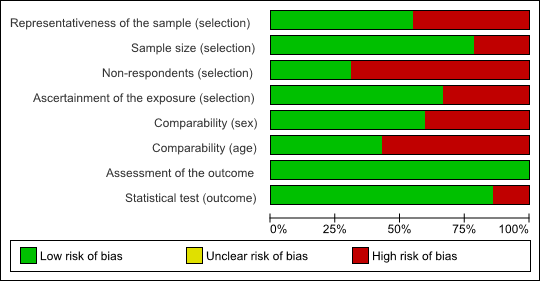


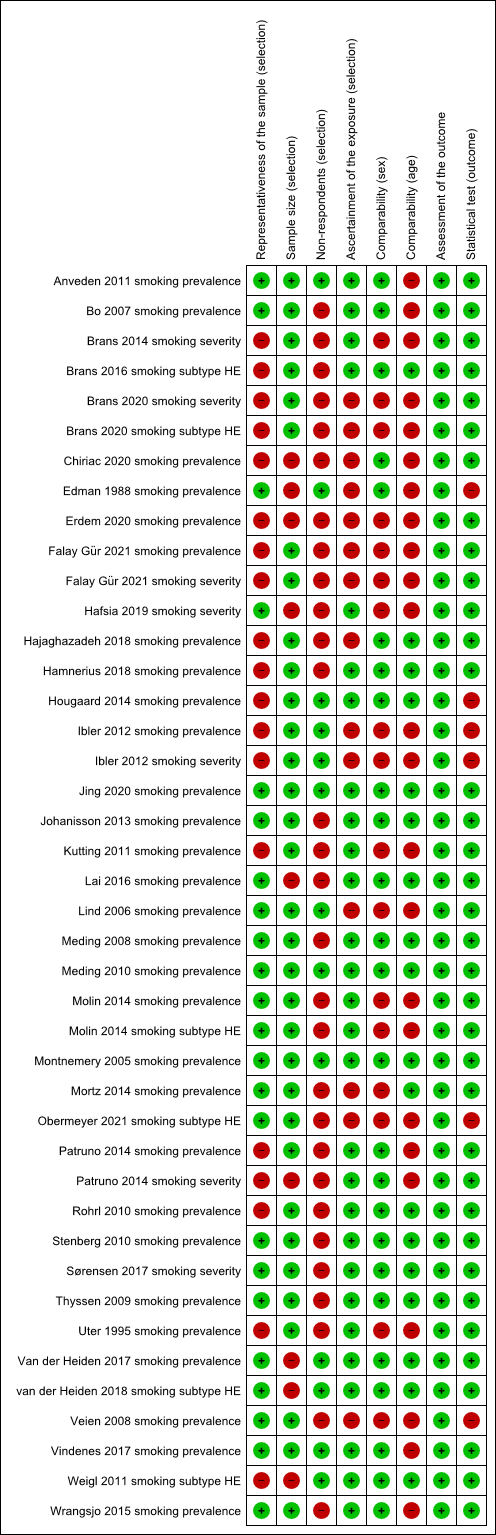
Risk of bias summary 1: review authors' judgements about each risk of bias item for each included cross-sectional study outcome for smoking and hand eczema (prevalence, subtype and severity, n=42) .

Risk of bias graph 2: review authors' judgements about each risk of bias item presented as percentages across all included cohort study outcomes for smoking and hand eczema (incidence and prognosis, n=5).


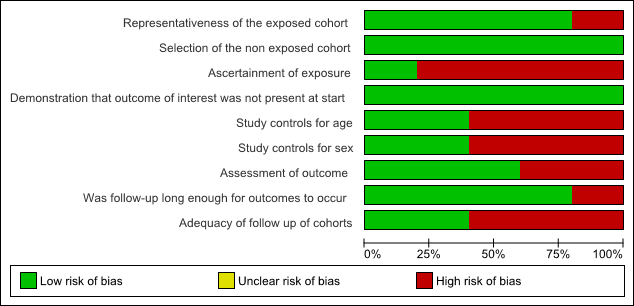


Risk of bias summary 2: review authors' judgments about each risk of bias item for each included cohort study outcomes for smoking and hand eczema (incidence and prognosis, n=5).


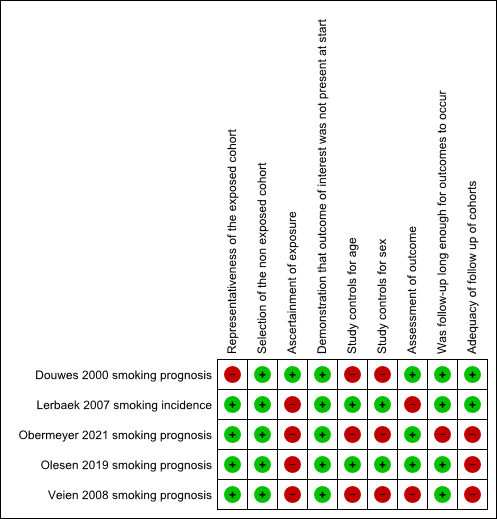


Risk of bias graph 3: review authors' judgments about each risk of bias item presented as percentages across the included case-control study outcome for smoking and prevalence of hand eczema (n=1).


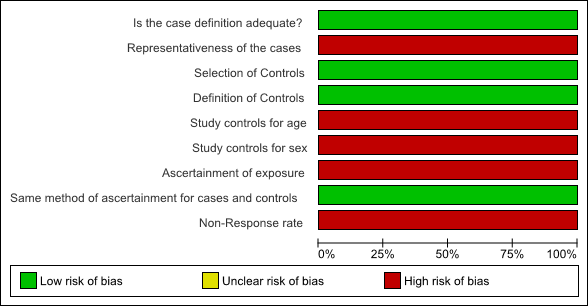


Risk of bias summary 3: review authors' judgements about each risk of bias item for the included case-control study outcome for smoking and prevalence of hand eczema (n=1).


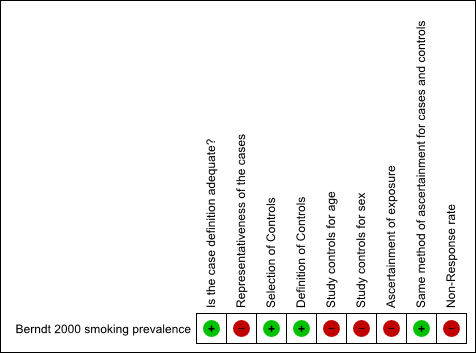


***Stress***

Risk of bias graph 4: review authors' judgments about each risk of bias item presented as percentages across all included cross-sectional study outcomes for stress and hand eczema (prevalence and severity, n=12).


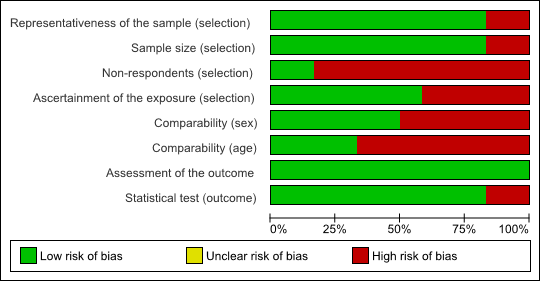


Risk of bias summary 4: review authors' judgements about each risk of bias item for each included cross-sectional study outcomes for stress and hand eczema (prevalence and severity, n=12).


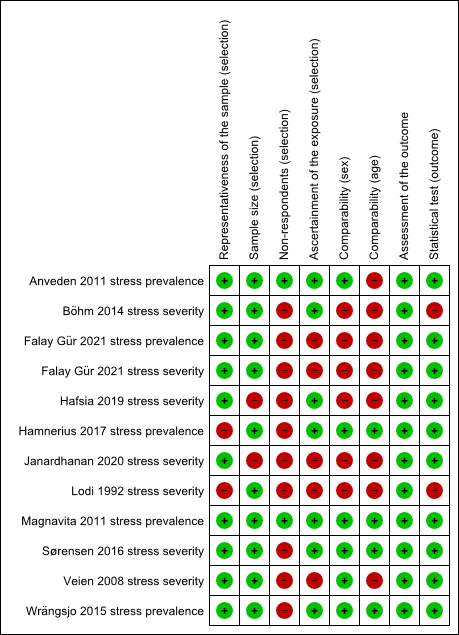


Risk of bias graph 5: review authors' judgements about each risk of bias item presented as percentages for the included case-control study outcome for stress and prevalence of hand eczema (n=1).

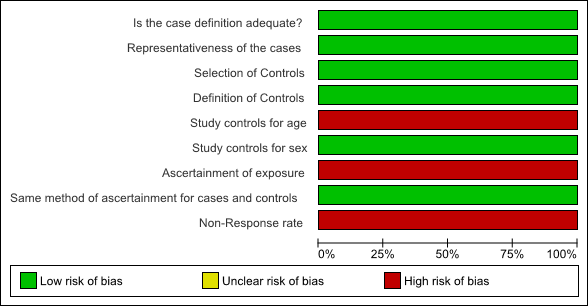


Risk of bias summary 5: review authors' judgements about each risk of bias item for the included case-control study outcome for stress and prevalence of hand eczema (n=1).


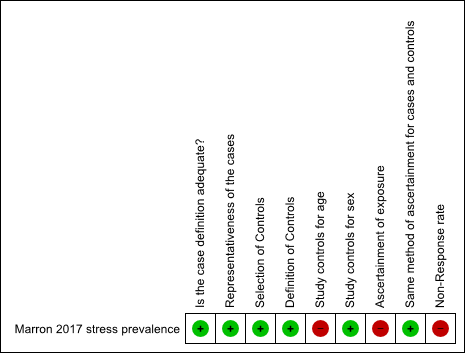


Risk of bias graph 6: review authors' judgments about each risk of bias item presented as percentages across the included cohort study outcome for stress and prognosis of hand eczema (n=1).


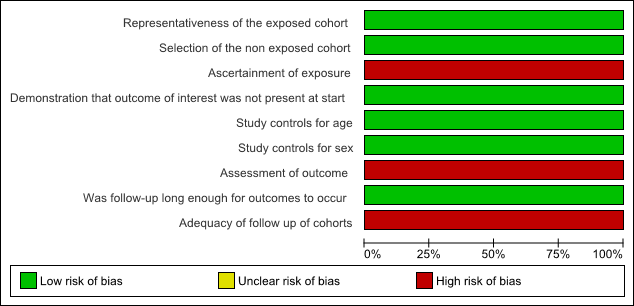


Risk of bias summary 6: review authors' judgments about each risk of bias item for the included cohort study outcome for stress and prognosis of hand eczema.


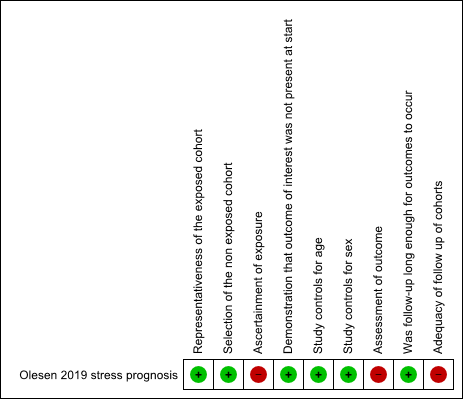


***Body Mass Index (BMI)***

Risk of bias graph 7: review authors' judgments about each risk of bias item presented as percentages across all included cross-sectional study outcomes for BMI and hand eczema (prevalence, subtype and severity, n=11).


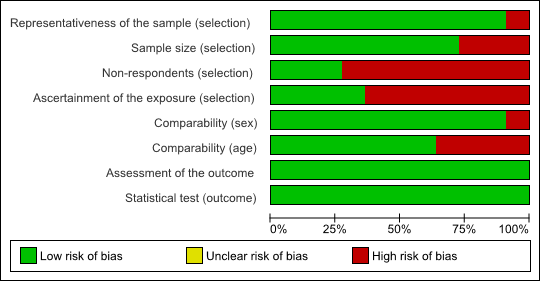


Risk of bias summary 7: review authors' judgments about each risk of bias item for each included cross-sectional study outcome for BMI and hand eczema (prevalence, subtype and severity, n=11).


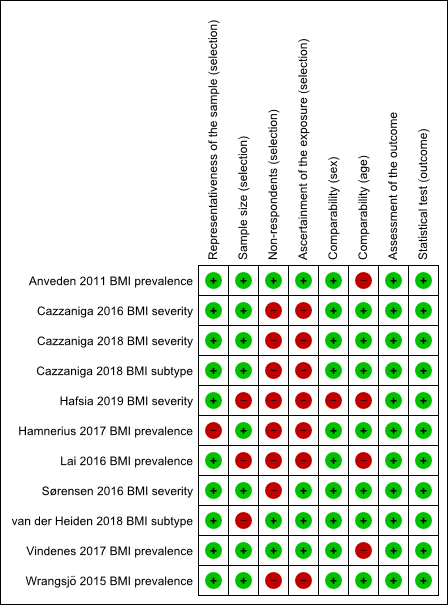


Risk of bias graph 8: review authors' judgments about each risk of bias item presented as percentages across the included cohort study outcome for BMI and prognosis of hand eczema (n=2).
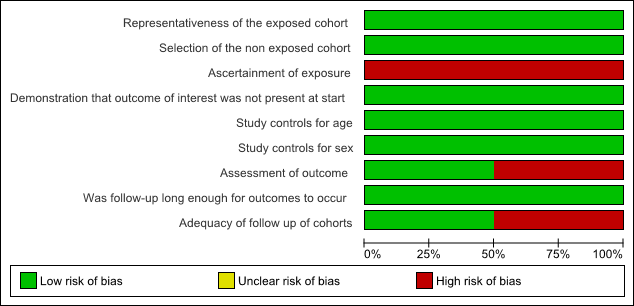


Risk of bias summary 8: review authors' judgments about each risk of bias item for the included cohort study outcome for BMI and prognosis of hand eczema (n=2).
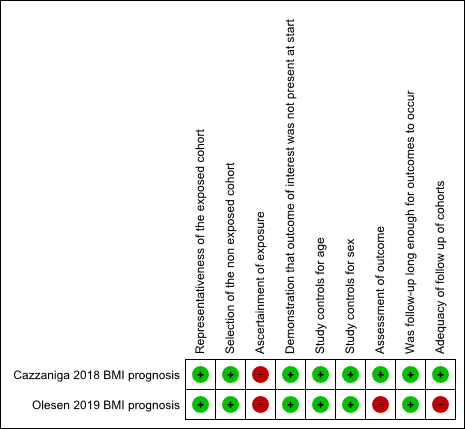


***Physical activity***

Risk of bias graph 9: review authors' judgements about each risk of bias item presented as percentages across all included cross-sectional study outcomes for physical activity and hand eczema (prevalence, subtype and severity, n=10).
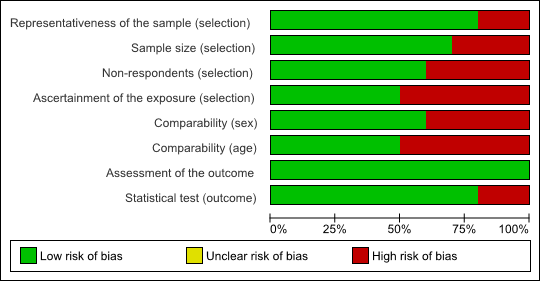


Risk of bias summary 9: review authors' judgements about each risk of bias item for each included cross-sectional study outcome for physical activity and hand eczema (prevalence, subtype and severity, n=10).


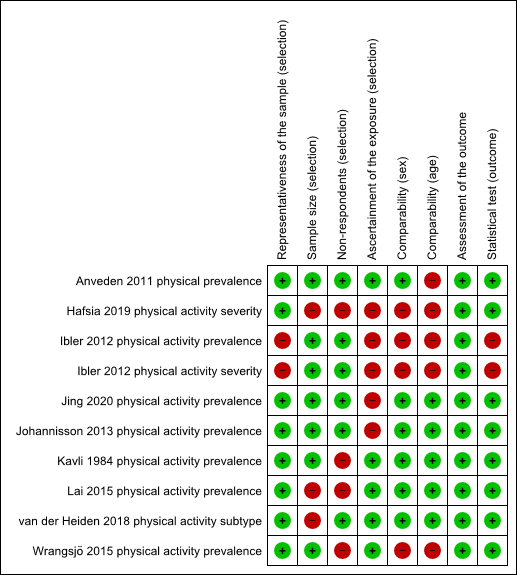


Risk of bias graph 10: review authors' judgements about each risk of bias item presented as percentages across the included cohort study outcome for physical activity and prognosis of hand eczema (n=1).
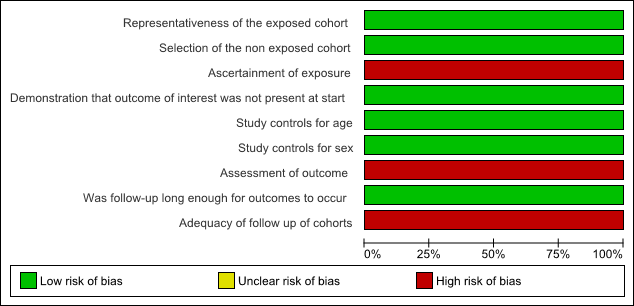


Risk of bias summary 10: review authors' judgements about each risk of bias item for the included cohort study outcome for physical activity and prognosis of hand eczema (n=1).


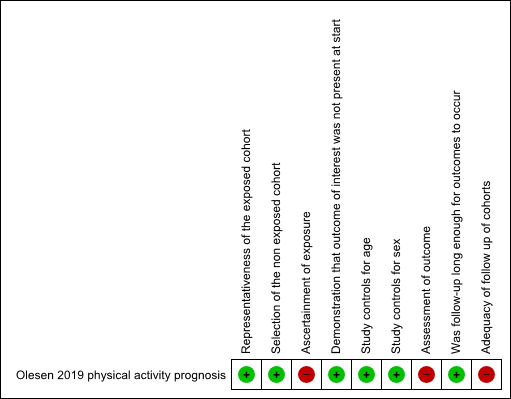


***Alcohol consumption***

Risk of bias graph 11: review authors' judgements about each risk of bias item presented as percentages across all included cross-sectional study outcomes for alcohol consumption and hand eczema (prevalence and severity, n=7).
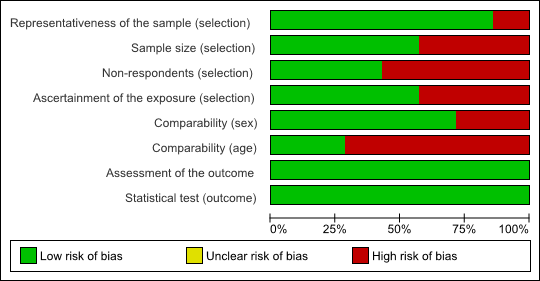


Risk of bias summary 11: review authors' judgements about each risk of bias item for each included cross-sectional study outcomes for alcohol consumption and hand eczema (prevalence and severity, n=7).


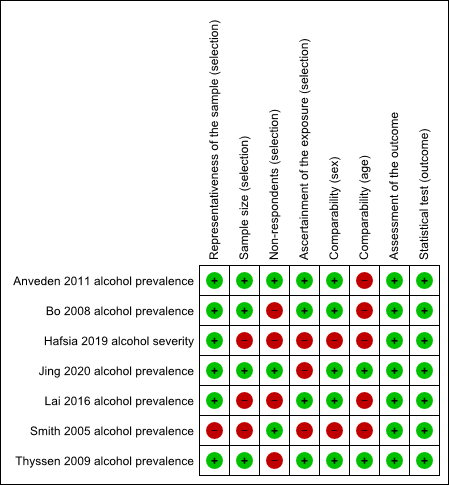


Risk of bias graph 12: review authors' judgements about each risk of bias item presented as percentages across the included cohort study outcome for alcohol consumption and incidence of hand eczema (n=1).
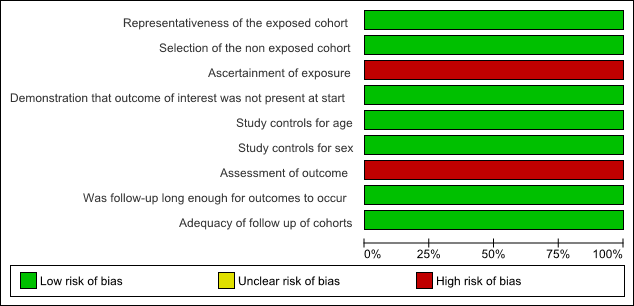


Risk of bias summary 12: review authors' judgements about each risk of bias item for the included cohort study outcome for alcohol consumption and incidence of hand eczema (n=1).


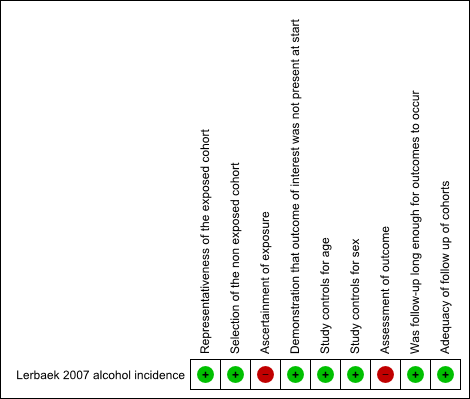


***Diet***

Risk of bias graph 13: review authors' judgements about each risk of bias item presented as percentages across the included cross-sectional study outcome for diet and prevalence of hand eczema (n=1).
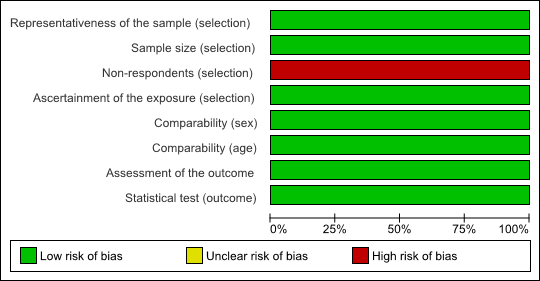


Risk of bias summary 13: review authors' judgements about each risk of bias item for the included cross-sectional study outcome for diet and prevalence of hand eczema (n=1).
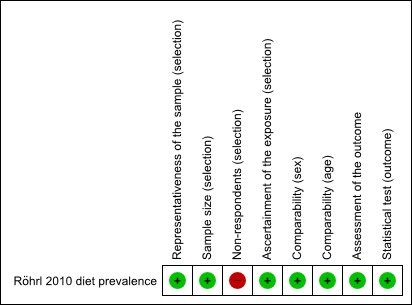


Risk of bias graph 14: review authors' judgements about each risk of bias item presented as percentages across the included cohort study outcome for diet and severity and prognosis of hand eczema (n=2).
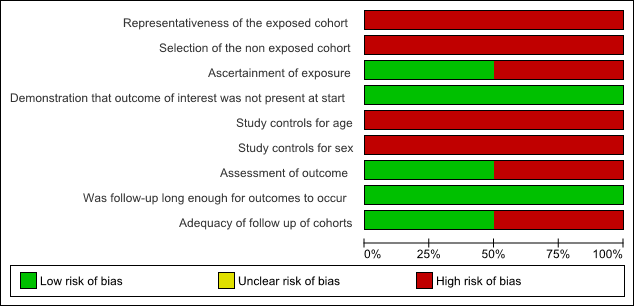


Risk of bias summary 14: review authors' judgements about each risk of bias item for the included cohort study outcome for diet and severity and prognosis of hand eczema (n=2).
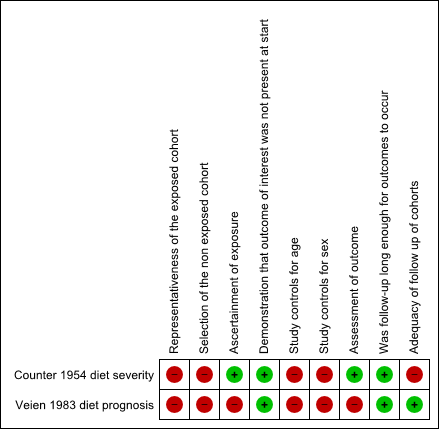


**Figure S4: Forest plot smoking and prevalence of hand eczema, sub-analysis only including study outcomes with a risk of bias score ≥ 7.**
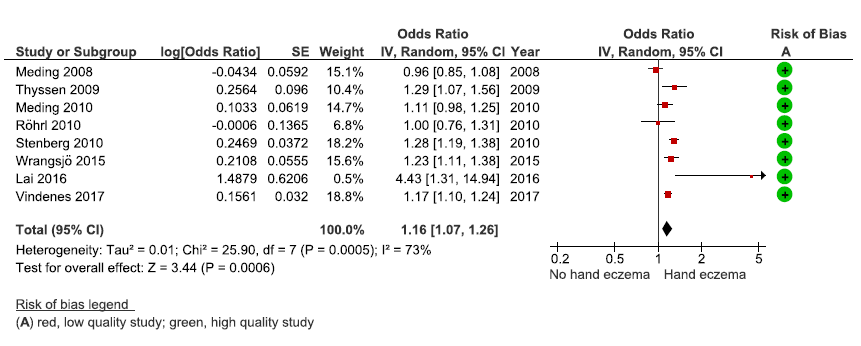


Legend: Cohort and case-control study outcomes with ≥ 6 points on the NOS, and cross-sectional study outcomes with ≥7 points, were considered low risk of bias.
Abbreviations: CI, confidence interval; df, degrees of freedom.

**Figure S5: Forest plot smoking and occurrence of hand eczema, only including study outcomes in a non-occupational setting.**
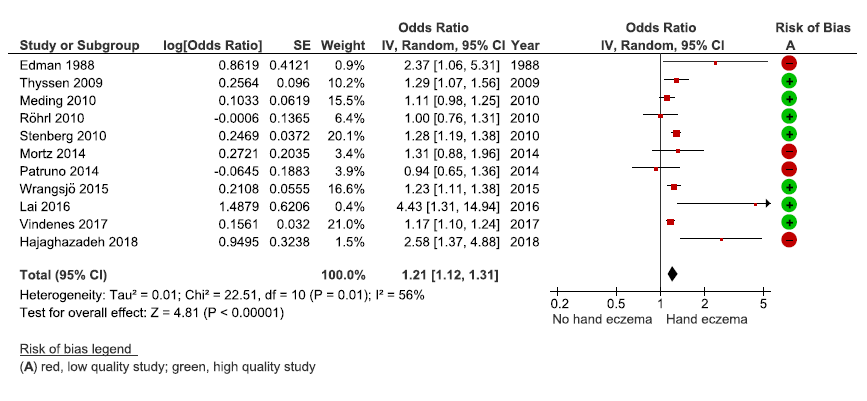


Legend: Cohort and case-control study outcomes with ≥ 6 points on the NOS, and cross-sectional study outcomes with ≥7 points, were considered low risk of bias.
Abbreviations: CI, confidence interval; df, degrees of freedom.

**Figure S6: Forest plot smoking and occurrence of hand eczema, subanalysis only including study outcomes in an occupational setting.**
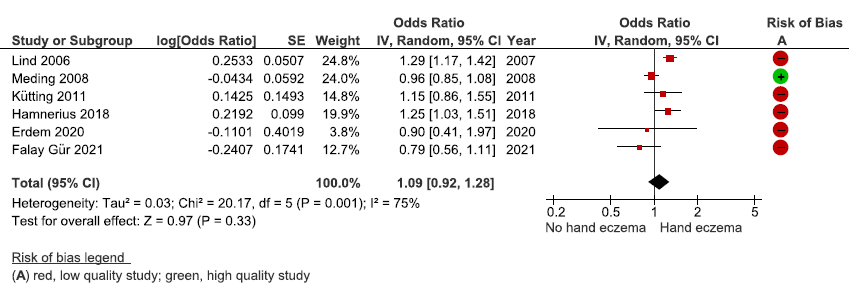


Legend: Cohort and case-control study outcomes with ≥ 6 points on the NOS, and cross-sectional study outcomes with ≥7 points, were considered low risk of bias.
Abbreviations: CI, confidence interval; df, degrees of freedom.

**Figure S7: Funnel plot smoking and prevalence of hand eczema. Including all studies from the meta-analysis.**


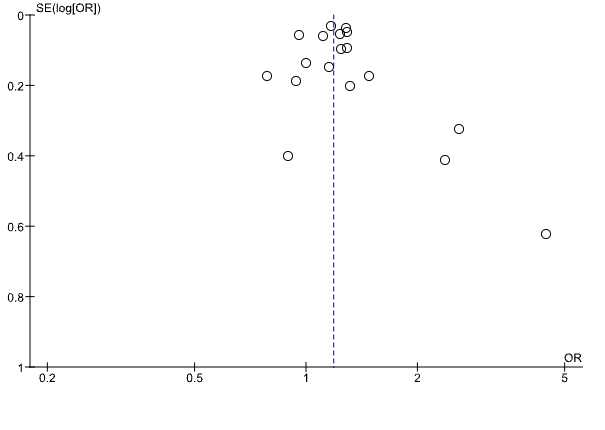


Abbreviations: OR, odds ratio; SE, standard error.

**Figure S8: Funnel plot smoking and prevalence of hand eczema. Including all studies from the sub-analysis of study outcomes with a risk of bias score ≥ 7.**


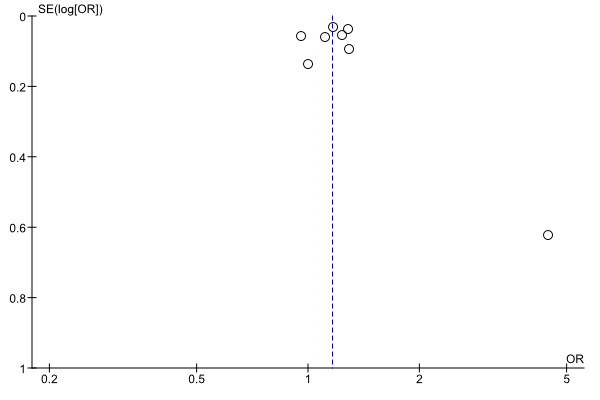


Abbreviations: OR, odds ratio; SE, standard error.

**References of excluded studies (from Table S2)**

1. Agner, T. *et al.* Contact sensitisation in hand eczema patients-relation to subdiagnosis, severity and quality of life: a multi-centre study. *Contact Dermatitis* **61**, 291–296 (2009).

2. Agner, T. [Hand eczema--a diagnostic and therapeutic challenge]. *Ugeskr. Laeger* **162**, 6850–6852 (2000).

3. Ahmed, A., Shah, R., Papadopoulos, L. & Bewley, A. An ethnographic study into the psychological impact and adaptive mechanisms of living with hand eczema. *Clin. Exp. Dermatol.* **40**, 495–501 (2015).

4. Ahmed, A. S. & Eldahshan, R. M. Occupational dermatoses: knowledge, attitudes and perceptions among motor vehicle repair workers. *Int. J. Occup. Saf. Ergon.* 1–7 (2021). doi:10.1080/10803548.2021.1962640

5. Ali, R., Aldraibi, M. & Abdulghani, M. M. Determination of the most frequent dermatosis and its associated risk factors in our society. *J. Dermatol. Nurses. Assoc.* **12**, (2020).

6. Alluhayyan, O. B. *et al.* Occupational-Related Contact Dermatitis: Prevalence and Risk Factors Among Healthcare Workers in the Al’Qassim Region, Saudi Arabia During the COVID-19 Pandemic. *Cureus* **12**, e10975 (2020).

7. Alsaidan, M. S. *et al.* The Prevalence and Determinants of Hand and Face Dermatitis during COVID-19 Pandemic: A Population-Based Survey. *Dermatol. Res. Pract.* **2020**, 6627472 (2020).

8. Apfelbacher, C. *et al.* Self-assessed disease severity is a powerful predictor of health-related quality of life in chronic hand eczema. *Exp. Dermatol.* **22**, e23 (2013).

9. Apfelbacher, C. *et al.* Patient-assessed disease severity is a strong determinant of health-related quality of life in chronic hand eczema. *J. Invest. Dermatol.* **133**, S101 (2013).

10. Arrandale, V. H., Kudla, I. & Holness, D. L. Smoking and allergic contact dermatitis: Causation or correlation? *Occup. Environ. Med.* **71**, A112 (2014).

11. Attwa, E. & el-Laithy, N. Contact dermatitis in car repair workers. *J. Eur. Acad. Dermatol. Venereol.* **23**, 138–145 (2009).

12. Aydin, A. İ., Atak, M., Özyazicioğlu, N. & Dalkizan, V. Determining the Frequency of and Factors in Hand Dermatitis among Nurses during the COVID-19 Pandemic. *Adv. Skin Wound Care* (2021). doi:10.1097/01.ASW.0000765916.20726.41

13. Babić, Ž., Samardžić, T. & Macan, J. Comparison of beautician and hairdressing apprentices with regard to skin health and skin barrier function. *Arh. Hig. Rada Toksikol.* **71**, 190–196 (2020).

14. Becker, J. Increased incidence of dermatoses in nicotine and alcohol abuse. *Hautartz* **46**, 735 (1995).

15. Bewley, A. & Walker, S. An ethnographic insight into the psychological impact of hand eczema. *J. Am. Acad. Dermatol.* **68**, AB74 (2013).

16. Koscec Bjelajac, A., Franic Zrinka, Ticic, M., Beuk, P. & Macan, J. Changes in sleep patterns in relation to hand eczema and atopy symptoms during the first year of hairdressing apprentices’ education. *J. Sleep Res.* 278–278 (2020).

17. Boehm, D. *et al.* Anxiety, depression and impaired health-related quality of life in patients with occupational hand eczema. *Contact Dermatitis* **67**, 184–192 (2012).

18. Brans, R. & John, S. M. Individual risk factors for occupational hand eczema. *Aktuelle Derm.* **41**, 20–24 (2015).

19. Brans, R., Hübner, A., Gediga, G. & John, S. M. Prevalence of foot eczema and associated occupational and non-occupational factors in patients with hand eczema. *Contact Dermatitis* **73**, 100–107 (2015).

20. Celik, V. & Ozkars, M. Y. An overlooked risk for healthcare workers amid COVID-19: Occupational hand eczema. *North. Clin. Istanbul* **7**, 527–533 (2020).

21. Chen, C.-J. *et al.* The total body burden of chromium associated with skin disease and smoking among cement workers. *Sci. Total Environ.* **391**, 76–81 (2008).

22. Chen, Y.-X., Cheng, H.-Y. & Li, L.-F. Prevalence and risk factors of contact dermatitis among clothing manufacturing employees in Beijing: A cross-sectional study. *Medicine (Baltimore).* **96**, e6356 (2017).

23. Cheon, J. J. *et al.* Evaluation of the dermatologic life quality among cleanroom workers in a secondary battery factory. *Ann. Occup. Environ. Med.* **28**, 39 (2016).

24. Chiriac, A. E. M., Matei, M., Stefanescu, C., Taranu, T. & Azoicai, D. Epidemiological study: Self-reported (work-related and occupational) hand eczema. *Allergy Eur. J. Allergy Clin. Immunol.* **75**, 466 (2020).

25. Chirico, F., Menchinelli, C., Giannandrea, F., Mazzarella, D. & Brandi, G. Stress-strain at work in hairdressers suffering from allergies and irritative diseases. *G. Ital. Med. Lav. Ergon.* **26**, 194–195 (2004).

26. Chou, T.-C., Wang, P.-C., Wu, J.-D. & Sheu, S.-C. Chromium-induced skin damage among Taiwanese cement workers. *Toxicol. Ind. Health* **32**, 1745–1751 (2016).

27. Collier, H. Pomplholyx and dietary deficiency. *Lancet* **268**, 200–201 (1956).

28. Cvetkovski, R. S. *et al.* Quality of life and depression in a population of occupational hand eczema patients. *Contact Dermatitis* **54**, 106–111 (2006).

29. Dalgard, F. J. *et al.* The psychological burden of skin diseases: a cross-sectional multicenter study among dermatological out-patients in 13 European countries. *J. Invest. Dermatol.* **135**, 984–991 (2015).

30. Devold, J. A. & Furberg, A.-S. Atopic disease in relation to Staphylococcus aureus carriage and spa type distribution in a subarctic adult population. *Nor. Epidemiol.* **28**, 26 (2019).

31. Dietz, J. B. *et al.* Incidence rates of occupational contact dermatitis in Denmark between 2007 and 2018: A population-based study. *Contact Dermatitis* **85**, 421–428 (2021).

32. Doğan, E. İ. & Kurt, B. Ö. New-onset or Exacerbated Occupational Hand Eczema among Healthcare Workers During the COVID-19 Pandemic: A Growing Health Problem. *Acta Dermatovenerol. Croat.* **291**, 21–29 (2021).

33. Dotterud, L. K. & Smith-Sivertsen, T. Allergic contact sensitization in the general adult population: a population-based study from Northern Norway. *Contact Dermatitis* **56**, 10–15 (2007).

34. Drewitz, K. P., Stark, K. J., Zimmermann, M. E., Heid, I. M. & Apfelbacher, C. J. Frequency of hand eczema in the elderly: Cross-sectional findings from the German AugUR study. *Contact Dermatitis* **85**, 489–493 (2021).

35. Fei, F. *et al.* The present situation investigation and possible factors of skin diseases in Southern Theater Command. *Med. J. Chinese People’s Lib. Army* **44**, 701–705 (2019).

36. Fors, R. *et al.* Lifestyle and nickel allergy in a Swedish adolescent population: effects of piercing, tattooing and orthodontic appliances. *Acta Derm. Venereol.* **92**, 664–668 (2012).

37. Gallicchio, L., Miller, S. R., Greene, T., Zacur, H. & Flaws, J. A. Adverse health outcomes among cosmetologists and noncosmetologists in the Reproductive Outcomes of Salon Employees (ROSE) study. *J. Toxicol. Environ. Health. A* **74**, 52–61 (2011).

38. Grabenhenrich, L. *et al.* Eczema in the first 20 years of life: Results from the multicenter allergy study-90 birth cohort. *Allergy Eur. J. Allergy Clin. Immunol.* **66**, 18 (2011).

39. Holm, J. G., Agner, T., Clausen, M.-L. & Thomsen, S. F. Quality of life and disease severity in patients with atopic dermatitis. *J. Eur. Acad. Dermatol. Venereol.* **30**, 1760–1767 (2016).

40. Hougaard, M. G., Winther, L., Zachariae, C., Søsted, H. & Johansen, J. D. Development of skin diseases in Danish hairdressing apprentices. *Contact Dermatitis* **70**, 61 (2014).

41. Huang, D. *et al.* Hand eczema among healthcare workers in Guangzhou City: a cross-sectional study. *Ann. Transl. Med.* **8**, 1664 (2020).

42. Kashyap, G., Singh, S. K. & Chauhan, B. Prevalence and Risk Factor of Occupational Skin Complaints among Male Tannery Workers of Kanpur, India. *Indian J. Dermatol.* **66**, 347–351 (2021).

43. Kendziora, B. *et al.* Evaluation of hand hygiene and onset of hand eczema after the outbreak of SARS-CoV-2 in Munich. *Eur. J. Dermatol.* **30**, 668–673 (2020).

44. Khalili, M. I., Dhaise, B. A., Abbossi, M. M. & Alwash, R. H. Contact dermatitis in auto repair workers. *Saudi Med. J.* **20**, 236–240 (1999).

45. Lee, C. H. *et al.* Lifetime exposure to cigarette smoking and the development of adult-onset atopic dermatitis. *Br. J. Dermatol.* **164**, 483–489 (2011).

46. Liang, Y., Zhang, X., Zhu, J., Ali, F. & Mo, H. Analysis of patch testing in patients with hand eczema at Shenzhen from 2016 to 2019. *Postep. dermatologii i Alergol.* **38**, 170–172 (2021).

47. Linnainmaa, M., Susitaival, P., Mäkelä, P. & Sjöblom, T. Respiratory symptoms and dermatoses among grinders and brazers of hard metal and stellite blades. *Occup. Med. (Chic. Ill).* **47**, 33–39 (1997).

48. Lysdal, S. H., Mosbech, H., Johansen, J. D. & Søsted, H. Asthma and respiratory symptoms among hairdressers in Denmark: results from a register based questionnaire study. *Am. J. Ind. Med.* **57**, 1368–1376 (2014).

49. Maarouf, M., Tran, K., Hendricks, A. J. & Shi, V. Pain burden in atopic dermatitis. *J. Invest. Dermatol.* **138**, S49 (2018).

50. McArthur, G. & Firkin, B. G. Smoking--another cause of palmar erythema? *The Medical journal of Australia* **156**, 71 (1992).

51. Metin, N., Turan, Ç. & Utlu, Z. Changes in dermatological complaints among healthcare professionals during the COVID-19 outbreak in Turkey. *Acta dermatovenerologica Alpina, Pannonica, Adriat.* **29**, 115–122 (2020).

52. Moldovan, H. R. *et al.* Prevention of Hand Eczema among Nurse Apprentice (PREVEDERM): An Interventional Study. *Ann. Work Expo. Heal.* **65**, 167–175 (2021).

53. Mortz, C. G., Bindslev-Jensen, C. & Andersen, K. E. Contact allergy and hand eczema in the TOACS cohort. *Contact Dermatitis* **70**, 35 (2014).

54. Murota, H., Kijima, A., Takihara, K. & Katayama, I. Research on the actual therapeutic situation in Japanese university students with atopic dermatitis. *J. Dermatol.* **39**, 16 (2012).

55. Nakamoto, M. *et al.* Soy product and isoflavone intake associations with allergic diseases in Japanese workers: rhinitis, dermatitis and asthma. *Asia Pac. J. Clin. Nutr.* **27**, 1277–1285 (2018).

56. Niemeier, V., Nippesen, M., Kupfer, J., Schill, W.-B. & Gieler, U. Psychological factors associated with hand dermatoses: which subgroup needs additional psychological care? *Br. J. Dermatol.* **146**, 1031–1037 (2002).

57. Nørreslet, L. B. *et al.* Impact of hand eczema on quality of life: metropolitan versus non-metropolitan areas. *Contact Dermatitis* **78**, 348–354 (2018).

58. O’Neill, H. *et al.* Occupational dermatoses during the COVID-19 pandemic: a multicentre audit in the UK and Ireland. *The British journal of dermatology* **184**, 575–577 (2021).

59. Patruno, C., Nisticò, S. P., Fabbrocini, G. & Napolitano, M. COVID-19, quarantine, and atopic dermatitis. *Medical hypotheses* **143**, 109852 (2020).

60. Pourani, M. R. & Abdollahimajd, F. Assessment of the psychosocial effects of hand dermatitis among healthcare workers during the COVID-19 pandemic. *Iran. J. Dermatology* **24**, 127–131 (2021).

61. Pourani, M. R. *et al.* Self-Reported Hand Eczema: Assessment of Prevalence and Risk Factors in Health Care Versus Non-Health Care Workers During the COVID-19 Pandemic. *Dermat. contact, atopic, Occup. drug* **32**, e19–e21 (2021).

62. Riala, R., Heikkilä, P. & Kanerva, L. A questionnaire study of road pavers’ and roofers’ work-related skin symptoms and bitumen exposure. *Int. J. Dermatol.* **37**, 27–30 (1998).

63. Rizzi, A. *et al.* Occupational hand dermatitis web survey in a university hospital during COVID-19 pandemic: the SHIELD study. *Med. Lav.* **112**, 320–326 (2021).

64. Rönmark, E. P. *et al.* Eczema among adults: Prevalence, risk factors and relation to airway diseases. Results from a large-scale population survey in Sweden. *Br. J. Dermatol.* **166**, 1301–1308 (2012).

65. Rosén, K. Pustulosis palmoplantaris and chronic eczematous hand dermatitis. Treatment, epidermal Langerhans cells and association with thyroid disease. *Acta Derm. Venereol. Suppl. (Stockh).* **137**, 1–52 (1988).

66. Rystedt, I. & Nilsson, E. [Hazardous work and predictors of hand eczema--vocational guidance of patients with atopic allergy]. *Lakartidningen* **86**, 714–717 (1989).

67. Saha, M., Podder, I. & Das, A. Skin Damage Induced by Enhanced Protective Measures in Frontline Doctors During Covid-19 Pandemic: A Web-Based Descriptive Study. *Indian J. Dermatol.* **66**, 145–150 (2021).

68. Sato, K., Kusaka, Y., Suganuma, N., Nagasawa, S. & Deguchi, Y. Occupational allergy in medical doctors. *J. Occup. Health* **46**, 165–170 (2004).

69. Silverberg, J. I. & Greenland, P. Eczema and cardiovascular risk factors in 2 US adult population studies. *J. Allergy Clin. Immunol.* **135**, 721-728.e6 (2015).

70. Silverberg, J. I. *et al.* Phenotypical Differences of Childhood- and Adult-Onset Atopic Dermatitis. *J. allergy Clin. Immunol. Pract.* **6**, 1306–1312 (2018).

71. Simonsen, A. B. *et al.* Increased occurrence of hand eczema in young children following the Danish hand hygiene recommendations during the COVID-19 pandemic. *Contact Dermatitis* **84**, 144–152 (2021).

72. Singh, M. *et al.* Occupational dermatoses in healthcare workers deployed in COVID-19 centres. *Dermatitis* **32**, e48 (2021).

73. Singh, R.; Prabhu, S. S.; Balachandran, C.; Pai, K. Acquired palmoplantar keratodermas : A study of clinical, histopathological and patch test findings. *J. Pakistan Assoc. Dermatologists* **30**, 388–395 (2020).

74. Smith, D. R., Ohmura, K. & Yamagata, Z. Prevalence and correlates of hand dermatitis among nurses in a Japanese teaching hospital. *J. Epidemiol.* **13**, 157–161 (2003).

75. Smith, D. R., Smyth, W., Leggat, P. A. & Wang, R.-S. Prevalence of hand dermatitis among hospital nurses working in a tropical environment. *Aust. J. Adv. Nurs. a Q. Publ. R. Aust. Nurs. Fed.* **22**, 28–32 (2005).

76. Smith, D. R. *et al.* Hand dermatitis among Korean nursing students. *Int. J. Nurs. Pract.* **12**, 160–165 (2006).

77. Son, J. H., Chung, B. Y., Kim, H. O. & Park, C. W. Clinical Features of Atopic Dermatitis in Adults Are Different according to Onset. *J. Korean Med. Sci.* **32**, 1360–1366 (2017).

78. Svensson, A. C. *et al.* Cohort profile: The Stockholm Public Health Cohort. *Int. J. Epidemiol.* **42**, 1263–1272 (2013).

79. Szepietowski, J. C., Salomon, J., Nowicka, D., Soter, K. & Horanin, M. Psychological Distress in Hospital Staff with Self-Reported Hand Dermatitis. *Dermatology Psychosom. / Dermatologie und Psychosom.* **3**, 180–182 (2002).

80. Techasatian, L. *et al.* Hand Hygiene Habits and Prevalence of Hand Eczema During the COVID-19 Pandemic. *J. Prim. Care Community Health* **12**, 21501327211018012 (2021).

81. Thuesen, B. H. *et al.* Cohort Profile: the Health2006 cohort, research centre for prevention and health. *Int. J. Epidemiol.* **43**, 568–575 (2014).

82. Veien, N. K., Hattel, T., Justesen, O. & Nørholm, N. Oral challenge with balsam of Peru. *Contact Dermatitis* **12**, 104–107 (1985).

83. Veien, N. K., Hattel, T., Justesen, O. & Nørholm, A. Dermatitis induced or aggravated by selected foodstuffs. *Acta Derm. Venereol.* **67**, 133–138 (1987).

84. Veien, N. K., Hattel, T., Justesen, O. & Nørholm, A. Dietary restrictions in the treatment of adult patients with eczema. *Contact Dermatitis* **17**, 223–228 (1987).

85. Veien, N. K., Hattel, T., Justesen, O. & Nørholm, A. Oral challenge with metal salts. (II). Various types of eczema. *Contact Dermatitis* **9**, 407–410 (1983).

86. Veien, N. K., hattel, T., Justesen, O. & Norholm, A. Dermatoses in coffee drinkers. *Cutis* **40**, 421–422 (1987).

87. von Manteuffel, L. [Hand eczema is preventable. A ‘thicker skin’ helps to achieve healthy skin]. *Kinderkrankenschwester Organ der Sekt. Kinderkrankenpfl.* **24**, 514–515 (2005).

88. Weistenhöfer, W., Uter, W., Bernet, F. & Drexler, H. The tissue viability imaging system-Suitable method for discovering minimal skin changes in occupational screenings? Results of a cross-sectional field study. *Ski. Res. Technol. Off. J. Int. Soc. Bioeng. Ski. [and] Int. Soc. Digit. Imaging Ski. [and] Int. Soc. Ski. Imaging* **25**, 553–563 (2019).

89. Xerfan, E. M. S. *et al.* Hand eczema, obsessive compulsive disorder and the role of sleep: a triad of associated factors and the influence of the current pandemic. *Sleep medicine* **79**, 164–165 (2021).

90. Yuan, Y. *et al.* Behavior of Nutritional Supplements Use in Association With Inflammatory Skin Diseases in Chinese College Students. *Front. Nutr.* **8**, 615462 (2021).

91. Zeerak, S. *et al.* Clinical Pattern and Patch Test Profile of Hand Eczema in Hospital Employees in a Tertiary Care Hospital of North India. *Indian Dermatol. Online J.* **12**, 72–77 (2021).
